# Cardiovascular precision medicine – A pharmacogenomic perspective

**DOI:** 10.1017/pcm.2023.17

**Published:** 2023-06-29

**Authors:** Sandosh Padmanabhan, Clea du Toit, Anna F. Dominiczak

**Affiliations:** BHF Glasgow Cardiovascular Research Centre, School of Cardiovascular and Metabolic Health, University of Glasgow, Glasgow, UK

**Keywords:** pharmacogenomics, cardiovascular, warfarin, clopidogrel, cytochrome-P-450

## Abstract

Precision medicine envisages the integration of an individual’s clinical and biological features obtained from laboratory tests, imaging, high-throughput omics and health records, to drive a personalised approach to diagnosis and treatment with a higher chance of success. As only up to half of patients respond to medication prescribed following the current one-size-fits-all treatment strategy, the need for a more personalised approach is evident. One of the routes to transforming healthcare through precision medicine is pharmacogenomics (PGx). Around 95% of the population is estimated to carry one or more actionable pharmacogenetic variants and over 75% of adults over 50 years old are on a prescription with a known PGx association. Whilst there are compelling examples of pharmacogenomic implementation in clinical practice, the case for cardiovascular PGx is still evolving. In this review, we shall summarise the current status of PGx in cardiovascular diseases and look at the key enablers and barriers to PGx implementation in clinical practice.

## Impact statement

Pharmacogenomics, the study of the effect of inherited or acquired genetic variation on differences in drug response or adverse effects. Around 95% of the population carry one or more actionable pharmacogenetic variants and over 75% of adults over 50 years old are on a prescription with a known PGx association. Pharmacogenomic evidence for cardiovascular drugs is growing along with emerging evidence for efficacy and cost-effectiveness. Successful pharmacogenomic implementation in healthcare requires strong scientific evidence, comprehensive and updated clinical guidelines, clinician champions and stakeholder engagement.

## Introduction

An average one-size-fits-all approach is the foundation of the existing general healthcare paradigm of therapeutic, and preventative interventions. Whilst this is a very practical and effective strategy, only 40–50% of patients respond to treatment in this all-comers approach prescribed as per current practice, indicating a large proportion of the population may be facing a deficit in addressing their medical needs (Collins and Varmus, 2015). This requirement for a transformation in the current paradigm of healthcare has motivated the emergence of precision medicine as a more targeted approach to treatment (Goldberger and Buxton, 2013; Schork, 2015). Precision medicine envisages an integration of an individual’s clinical and biological features obtained from laboratory tests, imaging, high-throughput omics and health records, to drive a personalised approach to diagnosis and treatment with a higher chance of success (Collins and Varmus, 2015). The anticipated benefits of the precision medicine approach for patients are quicker diagnosis and targeted treatment leading to higher treatment success with minimal to no adverse drug reactions (ADRs), with wider benefits in terms of decreased healthcare costs and increased economic productivity.

One of the routes to precision medicine is pharmacogenomics (PGx), the study of the effect of inherited or acquired genetic variation on drug absorption, distribution, metabolism and excretion (pharmacokinetics) or modification of drug target or biological pathways (pharmacodynamics) resulting in variations in drug response or adverse effects. Around 95% of the population carry one or more actionable pharmacogenetic variants and over 75% of adults over 50 years old are on a prescription with a known PGx association (Chanfreau-Coffinier et al., 2019; Heise et al., 2020; Zhou and Lauschke, 2022; Zhou et al., 2023). The U.S. Food and Drug Administration (FDA) lists around 499 drugs which have PGx biomarkers in the labelling, with around a 100 of them linked to data supporting PGx-guided therapeutic recommendations (FDA, 2023a, 2023b). PharmGKB (PharmGKB, 2023a, 2023b) and the Clinical Pharmacogenetics Implementation Consortium (CPIC) (Relling et al., 2020) publish evidence-based, peer-reviewed guidelines on applying PGx test results into actionable prescribing decisions. PharmGKB Level 1 genes or gene–drug combinations are considered pharmacogenomically significant and are linked to specific prescribing guidance. Similarly, CPIC Levels A and B indicate that genetic information should be considered before prescribing.

CPIC currently reports around 480 gene–drug interactions, including 93 gene–drug pairs (24 genes with 75 drugs) that are annotated with Level A evidence and prescription guidelines (Crews et al., 2014; Ramsey et al., 2014; Hicks et al., 2015; Bell et al., 2017; Johnson et al., 2017; Amstutz et al., 2018; Relling et al., 2019; CPIC, 2022). Although integration of PGx into routine clinical practice is not widespread, the recent PREPARE trial demonstrated both efficacy and feasibility of implementation of a 12 gene pharmacogenomic panel across diverse European health-care system organisations and settings (Swen et al., 2023) even if only limited to current CPIC Level A drugs (Chanfreau-Coffinier et al., 2019; Heise et al., 2020; Relling et al., 2020; Hicks et al., 2021; Pritchard et al., 2022). Only a small subset of the roughly 15% of medications that cite PGx information on their labels have actionable pharmacogenes (Ehmann et al., 2015; Mehta et al., 2020). Of the approximately 20,000 human genes, only 34 of them are considered clinically actionable with PGx (PharmGKB level 1) (PharmGKB, 2023a, 2023b). The majority of PGx-labelled agents are cancer therapies targeted for somatic mutations, rather than germline variants. Actionable germline PGx variants are present for around 7% of medications with CPIC Level A or B recommendations directing prescribing changes based on genotype (Relling et al., 2020).

## Pharmacogenomics

The broad clinical relevance of PGx is evident across the medical spectrum from improving treatment efficacy to avoiding ADRs. *CYP2D6* genotype guided optimisation of opioid analgesia resulted in a 30% reduction in pain intensity among 24% of patients (Smith et al., 2019). Antidepressant prescribing guided by PGx variants across eight genes (*CYP1A2, CYP2C9, CYP2C19, CYP3A4, CYP2B6, CYP2D6, HTR2A, SLC6A4*) in the Genomics Used to Improve DEpression Decisions (GUIDED) trial (Greden et al., 2019) showed improved response and remission rates in difficult-to-treat depression, but no difference between the study arms for symptom improvement (primary outcome). A trial in a predominantly white human immunodeficiency virus type 1 infected population showed 100% elimination of immunologically confirmed abacavir hypersensitivity syndrome in those randomised to pre-emptive HLA-B*57:01-guided abacavir initiation (Mallal et al., 2008). Similarly, pre-emptive *DPYD* genotype guided dosing reduced from 73% to 28% the risk of fluoropyrimidine toxicity and completely abolished fluoropyrimidine-related mortality (Deenen et al., 2016). Whilst these examples are compelling, the case for cardiovascular PGx is still evolving. In this review, we shall summarise the current status of PGx in cardiovascular diseases (CVDs) and look at the key enablers and barriers to PGx implementation in clinical practice.

## Warfarin

The coumarin derivatives (warfarin, acenocoumarol and phenprocoumon) are a mainstay of CVD therapy due to their crucial role in preventing or treating thromboembolism.

Coumarins inhibit vitamin K epoxide reductase complex subunit 1 (VKORC1) and thence clotting factors II, VII, IX and X to yield its pharmacological anticoagulant effect (Verhoef et al., 2014). Coumarins are racemic mixtures with one dominant pharmacological enantiomer. For warfarin, S-warfarin is 3–5 times more potent than R-warfarin and is preferentially metabolised by CYP2C9 (Kaminsky and Zhang, 1997). Warfarin is unique in that, unlike most other drugs, its dose titration is based on coagulation levels in response to treatment. Warfarin has a narrow therapeutic index and exceeding optimal anticoagulation (measured by the international normalised ratio, INR) increases the risk of bleeding, necessitating frequent monitoring and dose titration (Landefeld and Beyth, 1993). One study found hospitalisation due to bleeding and supra-therapeutic INRs was seen in 6–7% of patients prescribed warfarin (Hylek et al., 2007; Lau et al., 2017), while conversely, decreased time in the therapeutic INR range (TTR) was associated with increased ischaemic stroke, other thromboembolic events and mortality (Jones et al., 2005; Cancino et al., 2014).

There is substantial interpatient variability in warfarin response, with warfarin doses necessary to attain target INR ranging from <1 mg/day to >10 mg/day (stable dosing after loading dose) (Pokorney et al., 2015). Genetic variation accounts for 55–60% of this dose variability: *VKORC1* (∼25%), *CYP2C9* (∼15%), CYP4F2*3 (∼1–7%) (Zhou et al., 2023). Non-genetic factors collectively account for <20%: age, body mass index (BMI), smoking and drug interactions (Rost et al., 2004; Wadelius et al., 2009; Verhoef et al., 2014; Bourgeois et al., 2016).

The CYP2C9*2, *3, *5, *6, *8 and *11 alleles reduce clearance of the more active S-warfarin, thus decreasing dose requirements by 5–7 mg/week in those carrying *2, *8 and *11 alleles, and 14 mg/week reported for the *3 and *5 alleles. Consequently, these variants are also associated with increased risk of over-anticoagulation. The *2 and *3 alleles are common among Europeans, while the *5, *6, *8 and *11 alleles occur almost exclusively in African ancestry populations (Johnson et al., 2017; Zhou et al., 2023).

*VKORC1* regulatory variant c.−1639G>A (rs9923231) is associated with reduced *VKORC1* expression and lower warfarin dose requirements, with the −1,639 AA (high sensitivity) genotype more common among Asians and the −1,639 GG (reduced sensitivity) genotype more common among Africans (Limdi et al., 2010; Johnson et al., 2017; Zhou and Lauschke, 2022). Consequently, warfarin dose requirements are, respectively, lower and higher in Asian and African ancestry patients, respectively, as compared to Europeans (Limdi et al., 2010).

The CYP4F2 enzyme contributes to the variation in warfarin dose requirements not by metabolising warfarin, but rather by metabolising 75–90% of all vitamin K consumed by humans. Vitamin K_1_ reduction to vitamin K hydroquinone is critical to clotting factor activation. The *3 allele (rs2108622) is associated with reduced CYP4F2 activity resulting in higher concentrations of vitamin K1 and, consequently, higher warfarin dose requirements compared to the *1 allele, but this affects only European and Asian populations, with no impact on African ancestry individuals (Danese et al., 2019; Zhou and Lauschke, 2022).

While *VKORC1* and *CYP2C9* variants have emerged as the main genetic contributors to warfarin dose requirements in European and Asian ancestry populations (Cooper et al., 2008), the associations in African ancestry populations include single nucleotide polymorphisms (SNPs) in the chromosome 10 CYP2C cluster and in chromosome 6 upstream of *EPHA7* (Perera et al., 2013; De et al., 2018; Zhou and Lauschke, 2022).

### Validation of PGx-based warfarin dosing

The complexity of estimating initial warfarin dosing has been significantly diminished by the development of dosing algorithms, which take into account not only an individual’s clinical features (e.g., age, BMI and use of CYP2C9 inhibiting drugs), but also their genotype (*VKORC1* −1639G>A, CYP2C9*2 and CYP2C9*3 alleles) (Gage et al., 2008; International Warfarin Pharmacogenetics et al., 2009). However, CYP2C9*5, *6, *8, *11 and rs12777823 are not represented in the algorithms significantly reducing their utility in patients of African ancestry. The Gage algorithm incorporates CYP2C9*5, *6 and CYP4F2*3 allele (Gage et al., 2008; International Warfarin Pharmacogenetics et al., 2009).

Three large multi-site RCTs (EU-PACT, COAG and GIFT) have evaluated the efficacy of genotype-guided warfarin dosing (Kimmel et al., 2013; Pirmohamed et al., 2013; Gage et al., 2017) incorporating *VKORC1* −1639G>A and CYP2C9*2 and *3 variants in a PGx algorithm, with *CYP4F2* additionally included in the GIFT trial (Gage et al., 2017). The primary endpoint was TTR for the EU-PACT and COAG trials (Kimmel et al., 2013; Pirmohamed et al., 2013) and clinical outcomes for the GIFT trial (Gage et al., 2017). PGx-guided dosing showed significant improvement in the primary endpoints for EU-PACT and GIFT, but not COAG trials. EU-PACT (Pirmohamed et al., 2013) compared genotype-guided warfarin dosing on days 1–5 followed by routine practice to routine practice. At 12 weeks, TTR was 7% higher in the genotype-guided arm (67.4% vs. 60.3%, *P* < 0.001). Conversely, TTR was similar in both the genotype-guided and clinically guided dosing arms of the COAG trial (4-week TTR 45.2% vs. 45.4%) (Kimmel et al., 2013). In the GIFT trial (Gage et al., 2017), the primary composite endpoint (INR ≥ 4, 30-day major bleeding, 30-day mortality death, 60-day incident venous thromboembolism) was lower in the genotype-guided group (10.8% vs. 14.7%, *P* = 0.02). Participants included in both the EU-PACT and GIFT trials were predominantly European. Although 27% of the COAG trial participants were African American, only the *CYP2C9* alleles common in Caucasians (*2 and *3) were genotyped. Thus, all the three trials were blind to African ancestry-specific variants, and failure to account for these variants resulted in substantial warfarin overdosing in African American participants in the genotype-guided arm of COAG (Kimmel et al., 2013). The reason is that CYP2C9*5, *6, *8 or *11 allele (present in ~15% of patients of African ancestry) or rs12777823 A allele (>40% of patients) may be misclassified as normal metabolisers (e.g., *1/*1) and dosed accordingly (Drozda et al., 2015).

Patients with two or more *CYP2C9* or *VKORC1* variants are more prone to rapid INR surges and supratherapeutic anticoagulation at warfarin initiation. This may explain the differences between EU-PACT which used a loading dose and COAG which did not (Arwood et al., 2017).

### Clinical implementation of warfarin PGx

CYP2C9*2, *3, *5, *6, *8, *11, and *VKORC1* −1639G>A alleles (Pratt et al., 2020) are the minimum set of panel variants supported by cost-effectiveness data on the implementation of multigene genotype-guided warfarin dosing (Zhu et al., 2020). Both the FDA and Dutch Pharmacogenetics Working Group (DPWG) genotype-guided dosing recommendations are limited to just *VKORC1* −1639G>A and CYP2C9*2 and *3 alleles. CPIC, in contrast, provides African and non-African specific guidance, with the former requiring CYP2C9*5, *6, *8 and *11 genotypes, and the latter requiring on CYP2C9*2 and *3 and *VKORC1* genotypes (Johnson et al., 2017). Presence of CYP4F2*3 allele in non-African individuals results in a 5–10% dose increase. For those of African ancestry, rs12777823 variant, if available, results in an additional 15–30% dose reduction (Johnson et al., 2017).

## Clopidogrel

Antiplatelet therapy is a cornerstone of atherosclerotic CVD management involving aspirin or a P2Y_12_ receptor antagonist (clopidogrel, prasugrel and ticagrelor), either as single agent therapy for secondary prevention or dual agents after percutaneous coronary intervention (PCI) (Roffi et al., 2016; Ibanez et al., 2018). Prasugrel and ticagrelor are more potent P2Y_12_ receptor antagonists with an increased bleeding risk but are preferred over clopidogrel in high-risk cases (Wallentin et al., 2009). Genetic variation is partly responsible for the observed variability in effectiveness of antiplatelet therapy (Angiolillo et al., 2017). Assessment of platelet function status is time-consuming, lacks standard reference values and is hence not clinically feasible for tailoring antiplatelet therapy. The prospect of a genotype profile providing a measure of antiplatelet efficacy and thus predicting adverse cardiovascular outcomes makes a compelling case for the use of PGx to personalise treatment.

Clopidogrel, the most commonly prescribed antiplatelet drug, is a prodrug that undergoes a two-step transformation to its active metabolite which irreversibly inhibits platelet activation (Kazui et al., 2010). CYP2C19 is involved in both activation steps, and thus, plays a crucial role in the bioactivation process of clopidogrel (Sangkuhl et al., 2010). *CYP2C19* is highly polymorphic with alleles representing a range of metaboliser phenotypes (summarised in Table 1; Kazui et al., 2010; Sangkuhl et al., 2010; Scott et al., 2013; Pratt et al., 2018; Zhou and Lauschke, 2022; Zhou et al., 2023).

**Table 1.** *CYP2C19* allele dependent enzyme activity

*CYP2C19* allele	Enzyme activity	Homozygous	Heterozygous (with *1 or *17)
*1	Normal	EM	UM
*2	None	PM	IM
*3	None	PM	IM
*4	None	PM	IM
*5	None	PM	IM
*6	None	PM	IM
*7	None	PM	IM
*8	None	PM	IM
*9	Decreased	PM	IM
*10	Decreased	PM	IM
*17	Increased	UM	UM

Abbreviations: EM, extensive metaboliser; IM, intermediate metaboliser; PM, poor metaboliser; UM, ultrarapid metaboliser.

The CYP2C19 poor metaboliser (PM) and intermediate metaboliser (IM) phenotypes have higher on-treatment platelet reactivity and an increased risk of ischaemic events compared to the normal metaboliser (NM) phenotype (*1/*1 genotype) (Varenhorst et al., 2009; Mega et al., 2009a). The equivalent of a 75 mg dose of clopidogrel in NMs is 225 mg in IMs, but 300 mg is insufficient in PMs (Mega et al., 2011; Price et al., 2012; Carreras et al., 2016). The antiplatelet drugs prasugrel and ticagrelor are not affected by the *CYP2C19* genotype, offering the option for switching IMs and PMs to these drugs in preference to clopidogrel dose escalation in the absence of contraindications (Varenhorst et al., 2009; Mega et al., 2009b; Wallentin et al., 2010).

Several real-world studies showed a significantly higher risk of major adverse cardiovascular events (MACE) in CYP2C19 PMs and IMs compared to NMs (Hulot et al., 2010; Mega et al., 2010; Holmes et al., 2011; Zabalza et al., 2012; Sorich et al., 2014; Cavallari et al., 2018; Kheiri et al., 2020). However, the higher risk of MACE in clopidogrel-treated PMs and IMs was less evident in lower-risk populations, such as atrial fibrillation or medically managed acute coronary syndrome (ACS) cases (Bauer et al., 2011; Holmes et al., 2011). Two prospective trials, POPular Genetics (Claassens et al., 2019) and TAILOR-PCI (Pereira et al., 2020) trials stratified IMs and PMs to prasugrel or ticagrelor while NMs received clopidogrel. The *CYP2C19*-guided approach reduced bleeding risk and was non-inferior to treatment with prasugrel or ticagrelor in preventing atherothrombotic events in the POPular Genetics study that enrolled post ST-segment elevation MI patients undergoing PCI (Claassens et al., 2019). In the TAILOR-PCI trial (Pereira et al., 2020), patients with either stable coronary disease or ACS undergoing PCI showed lower rates of the composite cardiovascular primary endpoint in the genotype-guided group compared to the non-genotype-guided cohort at 1-year follow-up, but this did not reach statistical significance (HR 0.66; 95% CI 0.43–1.02; *P* = 0.06) (Pereira et al., 2020). A post hoc analysis indicated benefit in the genotype-directed group during the first 3 months after PCI (HR 0.21; 95% CI 0.08–0.54; *P* = 0.001) (Pereira et al., 2020). Other indications for clopidogrel include stroke prevention and peripheral arterial disease. PMs and IMs show reduced rates of stent patency after endovascular treatment for peripheral arterial disease (Guo et al., 2014; Diaz-Villamarin et al., 2016). For stroke, a large randomised controlled trial (RCT) showed that absence of the *CYP2C19* no-function allele in patients with a minor ischaemic stroke or transient ischaemic attack (TIA) predicted better effectiveness of clopidogrel plus aspirin over aspirin alone (Wang et al., 2016). A meta-analysis including nearly 5,000 clopidogrel-treated patients with ischaemic stroke or TIA confirmed higher risk of new stroke in PMs and IMs (Pan et al., 2017).

### Clinical implementation of clopidogrel PGx

Since 2010, the FDA, European Medicine Agency (EMA) and other regulatory bodies recommend alternative P2Y_12_ inhibitors to clopidogrel in PMs (but not IMs) in their labels (Holmes et al., 2010). The FDA table of gene–drug pairs includes therapeutic management recommendations for IMs and PMs (FDA, 2023a, 2023b), which is echoed by CPIC guidelines citing ‘strong’ evidence for IMs and PMs with ACS or undergoing PCI, and ‘moderate’ evidence PMs for all indications. In all of the above cases, alternative antiplatelet agents are recommended (Lee et al., 2022).

Joint PCI guidelines from 2016 by the American College of Cardiology (ACC) and the American Heart Association (AHA) recommend against routine genotyping for all patients undergoing PCI, but to consider testing high-risk patients and use either prasugrel or ticagrelor for patients with the no-function allele. The 2020 European Society of Cardiology (ESC) guidelines were influenced by the POPular Genetics trial to recommend genotype-guided de-escalation for post-PCI patients deemed to be at high bleeding risk (Claassens and Sibbing, 2020; Collet et al., 2021).

*CYP2C19*-guided antiplatelet therapy after PCI is one of the most common PGx tests in clinical practice (Empey et al., 2018) conducted either for patients at high risk of MACE in line with ACC/AHA guidelines or for all-comers (Empey et al., 2018). If point-of-care genotyping is not available, a de-escalation approach is proposed where patients are commenced on prasugrel or ticagrelor initially pending genotype results and then switched to clopidogrel if the genotype results indicate the NM phenotype. This approach maximises benefit given the high risk of atherothrombotic events early after ACS and PCI, while reducing the high risk of bleeding with prasugrel and ticagrelor during long-term therapy (Becker et al., 2011; Rollini et al., 2016; Angiolillo et al., 2017). The case for implementing pre-emptive *CYP2C19* genotyping (Peterson et al., 2016) is evident due to the impact of *CYP2C19* genotype on other drugs in addition to clopidogrel, such as proton pump inhibitors (Lima et al., 2021) and selective serotonin reuptake inhibitors (SSRIs) (Hicks et al., 2015).

## Direct-acting oral anti-coagulants

Apixaban, dabigatran, edoxaban and rivaroxaban are direct-acting oral anticoagulants (DOACs) with several advantages compared to warfarin – wider therapeutic index, regular monitoring not required, lower risk of intracranial haemorrhage, stroke or systemic embolic events (Proietti et al., 2018). Despite the favourable profile of DOACs, their higher cost, lower adherence rates, limited indications, and the high cost of reversal agents has limited uptake of DOAC compared to warfarin (Zhu et al., 2018; Ho et al., 2020). Pharmacokinetic variation related to genetic variation is indicated but there is no data on clinical outcomes yet.

In a sub-study of the ENGAGE AF TIMI-48 trial (which compared warfarin and edoxaban in atrial fibrillation patients; Mega et al., 2015) warfarin-treated participants with a sensitive or highly sensitive genotype (e.g., *VKORC1* −1639AA or CYP2C9*1/*3) spent a greater proportion of time within the supratherapeutic INR range (i.e., INR >4) and had higher rates of bleeding in the initial 90 days of treatment, as compared to those with non-sensitive genotypes. In a genetic sub-study of the RE-LY trial (dabigatran versus warfarin in atrial fibrillation), carriers of the *CES1* rs2244613 minor allele had a reduced risk of bleeding with dabigatran than with warfarin (Shi et al., 2016).

## Statins

Lipid lowering treatment by statins (HMG-CoA reductase inhibitors) are used in the prevention of CVD (Catapano et al., 2016). Statin-associated muscle symptoms (SAMS) (range from mild myalgia without an elevation in creatine kinase to life-threatening rhabdomyolysis or autoimmune-necrotizing myositis) are the commonest reasons for treatment discontinuation (Alfirevic et al., 2014). A number of enzymes and transporters are responsible for intracellular skeletal myocyte entry that underlie disruption of muscle function leading to SAMS (Turner and Pirmohamed, 2019). Hepatic uptake and elimination of statins are mainly carried out by the solute carrier anion transporter family 1B1 gene (*SLCO1B1*) encoding the organic anion transporting polypeptide 1B1 (OATP1B1) (Shitara, 2011). The rs4149056 SNP in the *SLCO1B1* gene (SLCO1B1*5) is linked to OATP1B1 function (Tirona et al., 2001) with the C allele being associated with decreased OATP1B1 transporter function with greatest reduction in homozygous patients resulting in significantly increased plasma concentrations of all statins, except fluvastatin (Tirona et al., 2001). Additionally, the risk of myopathy increases by 2.6 and 4.3 per copy of SLCO1B1*5 in patients, respectively, on simvastatin 40 mg and 80 mg daily (Tirona et al., 2001). The mechanism of SLCO1B1*5 variant causing statin-related myopathy is through the accumulation of circulating simvastatin acid (the active form of simvastatin) reflecting liver transport (Choi et al., 2015). This effect is most prominent for simvastatin followed by pitavastatin, lovastatin and atorvastatin (Ramsey et al., 2014). Each copy of the C allele of rs4149056 increases the risk of statin-induced myopathy threefold in genome-wide association studies (GWAS) (Carr et al., 2019). Atorvastatin is partially metabolised by the CYP3A and UDP-glucuronosyltransferase 1A1 (UGT1A) enzyme families. One study showed the SNP rs45446698 just upstream of *CYP3A7* and another, rs887829, located in multiple overlapping *UGT1A* genes, to be associated with atorvastatin-to-metabolite ratios in patients with ACS (Turner et al., 2020). Inconsistent associations with SAMS have been reported for polymorphisms in *CYP3A4*, *ABCB1*, *COQ2* (involved in coenzyme Q10 synthesis) and *GATM* (involved in creatine synthesis) (Fiegenbaum et al., 2005; Hoenig et al., 2011; Mangravite et al., 2013; Carr et al., 2019).

### Validation of PGx-based statin dosing

The pragmatic *SLCO1B1* genotype-informed statin therapy (GIST) trial randomised patients who had discontinued any statins due to myalgia to *SLCO1B1* genotype guided therapy (rosuvastatin, pravastatin, or fluvastatin for SLCO1B1*5 carriers and any statin for non-carriers) or standard care (Peyser et al., 2018). At the end of 8-month follow-up, increased statin re-initiation, reduced LDL-C levels, and no change in self-reported medication adherence were seen in those randomised to genotype guided (Peyser et al., 2018). Whilst these results are interesting, the inclusion of patients who developed myopathy from any statins in the trial limits the translational potential of the results. This is because the impact of *SLCO1B1* variation is highest for simvastatin and variable for other statins, hence the results of the trial do not present a clear case for genotype-guided simvastatin therapy.

### Clinical implementation of statin PGx

The SLCO1B1*5 variant (rs4149056) shows wide population differences (1%, 8% and 16% in African, Asian and European populations, respectively). CPIC recommends not exceeding a dose of simvastatin 20 mg/day or, prescribing another statin (rosuvastatin or pravastatin) in patients who carry at least one rs4149056 C allele (Voora et al., 2009; Danik et al., 2013; Ramsey et al., 2014; Lamoureux et al., 2017). The French National Network of Pharmacogenetics recommends commencing statins in patients with risk factors for myopathy only after rs4149056 genotyping (Lamoureux et al., 2017). The DPWG recommends that homozygotes avoid simvastatin entirely and individuals with other clinical risk factors for SAMS avoid atorvastatin (de Keyser et al., 2014; Bank et al., 2019; Linskey et al., 2020; Turner et al., 2020).

## Beta blockers

β-Adrenergic receptor antagonists, or beta blockers, are indicated for treatment of heart failure, hypertension, and secondary prevention of myocardial infarction. CYP2D6 is responsible for biotransformation of 70–80% of an oral dose of metoprolol and has negligible effects on other beta blockers (Ingelman-Sundberg et al., 2007; Baudhuin et al., 2010; Blake et al., 2013; Zisaki et al., 2015; Vieira et al., 2018). There is only weak evidence for PGx-guided prescribing of beta blockers (PharmGKB level 2–3, CPIC level B/C). Compared to EMs, IMs and PMs are associated with a decreased heart rate (Bijl et al., 2009; Batty et al., 2014; Anstensrud et al., 2020) and lower diastolic BP (Bijl et al., 2009; Batty et al., 2014; Hamadeh et al., 2014; Anstensrud et al., 2020). These studies have not studied the entire spectrum of major variations in *CYP2D6* and have not been independently validated.

Three other genes (*ADRB1*, *ADRB2* and *GRK5*) have been associated with the beta blocker pharmacodynamics rather than pharmacokinetics, but there is no evidence of clinical utility for using these variants to guide prescribing (White et al., 2003; Pacanowski et al., 2008; Magvanjav et al., 2017; Huang et al., 2018).

FDA and DPWG have slightly different recommendations on metoprolol dosing. The FDA recommends caution with co-administration of strong CYP2D6 inhibitors (SSRIs, antipsychotics) or substrates. The DPWG recommend cautious dose titration and reduced maximal doses in CYP2D6 IMs and PMs supramaximal metoprolol dose or an alternative beta blocker in UMs (Brouwer et al., 2022).

## Hydralazine

Hydralazine is a direct vasodilator seldom used in the treatment of hypertension (Whelton et al., 2018). Hydralazine is metabolised primarily by hepatic *N*-acetyltransferase type 2 (NAT2) acetylation. The common NAT2*4 genetic variant defines a ‘rapid acetylator’ phenotype with decreased hydralazine levels after drug administration (Gonzalez-Fierro et al., 2011; Han et al., 2019). Homozygous NAT2*5, *6, and *7 indicate a ‘slow acetylator’ phenotype, while heterozygous individuals (e.g., *4/*5) are ‘intermediate acetylators’. One study of resistant hypertension patients demonstrated that only those with the slow acetylator phenotype showed notable blood pressure reductions in response to hydralazine (Spinasse et al., 2014).

One of the rare side effects of hydralazine is the occurrence of lupus-like symptoms, with indirect evidence suggesting slow acetylators are more prone to developing this ADR (Weber and Hein, 1985; Mazari et al., 2007; Schoonen et al., 2010). However, clinical utility and cost-effectiveness data are lacking.

## Antiarrhythmic drugs

Inhibition of the rapid component of the delayed rectifier potassium current, *I*_kr_, encoded by *KCNH2* is the commonest cause of drug induced long QT syndrome (LQTS) and torsades des pointes (TdP; ventricular tachycardia (Roden and Viswanathan, 2005; Wada et al., 2022).

Similar to beta blockers, the class 1 antiarrhythmic drugs flecainide and propafenone are metabolised by CYP2D6 (PharmGKB level 2A, CPIC level B/C; Doki et al., 2015; Rouini and Afshar, 2017) with *CYP2D6* genotype-related differences in QTc interval (Lim et al., 2010). The FDA recommends caution in the use of propafenone in patients with CYP2D6 deficiency when combined with CYP3A4 inhibition. The DPWG recommends a dose reduction of 50% and 30%, respectively, for flecainide and propafenone in CYP2D6 PMs.

Quinidine- or dofetilide-induced QT prolongation and drug-induced TdP was significantly associated with a polygenic risk score constructed from 61 SNPs excluding the *CYP2D6* locus (Arking et al., 2014; Strauss et al., 2017). Though not validated, this highlights the potential for using polygenic risk scores in predicting drug-induced arrhythmias.

## PGx implementation

Successful pharmacogenomic implementation in healthcare require strong scientific evidence, comprehensive and updated clinical guidelines, clinician champions and stakeholder engagement (Manolio et al., 2013).

### Laboratory

**Figure 1.**
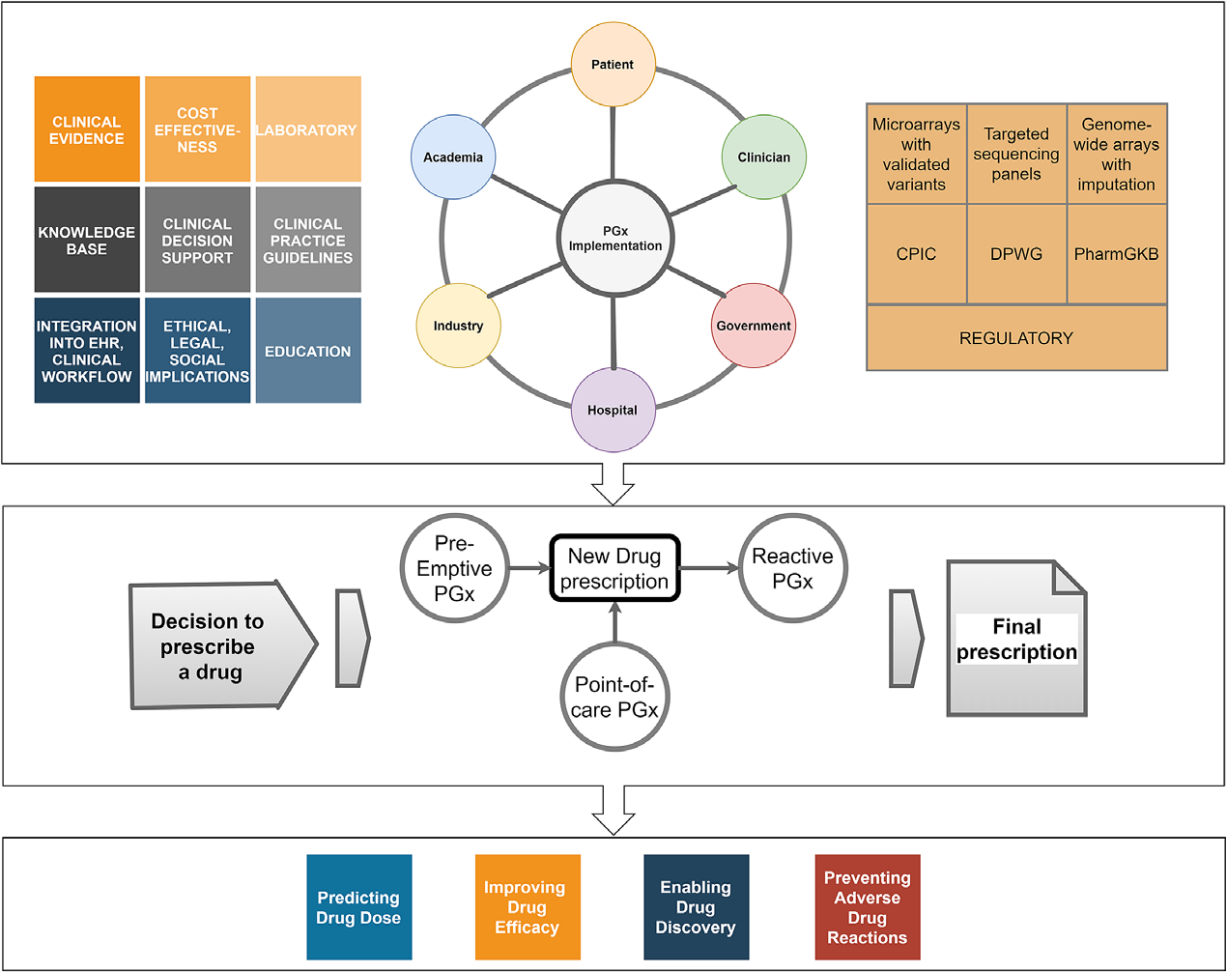
Pharmacogenomic implementation. The top panel shows the range of stakeholders, technology, knowledge and evidence that need to be harnessed to realise the value of PGx. The middle panel depicts the uses of PGx in the clinical prescribing pathway. The bottom panel presents the applications of PGx. CPIC, the Clinical Pharmacogenetics Implementation Consortium; DPWG, Dutch Pharmacogenetics Working Groups; PharmGKB, the Pharmacogenomics Knowledge Base.

Characterisation of pharmacogenomic variants in patients requires a certified molecular pathology laboratory to ensure analytical accuracy, precision, sensitivity and specificity of the results (Tayeh et al., 2022). Most clinical PGx tests based on selected panel of clinically relevant variants (single gene or multigene) are more cost-effective than sequencing panels. It is likely that the decreasing cost of sequencing will make sequencing cost-competitive over multi-gene panels in the future (Figure 1).

### Guidelines and clinical decision support systems

Effective pharmacogenomic guided prescribing requires evidence from multiple sources to be distilled into guidelines and made available through clinical decision support systems (CDSS) that distil information on drug–gene interactions from published guidelines or prescribing labels. Clinical Pharmacogenetics Implementation Consortium (CPIC) and the Dutch Pharmacogenetics Working Groups (DPWG) have published guidelines covering 66 medications across several drug classes. However, the major PGx guideline and recommendation sources are not completely concordant in terms of their advice. A recent study found inconsistencies in clinical PGx recommendations (48.1%) and in 93.3% of recommendations from CPIC, FDA and clinical practice guidelines (Shugg et al., 2020). These inconsistencies were spread across a range of domains – recommendation category (29.8%), the patient group (35.4%) and routine screening (15.2%), suggesting a potential barrier to rapid PGx implementation until this is resolved.

CDSS is an effective tool to guide clinicians with limited PGx knowledge (van der Wouden et al., 2017). In pre-emptive PGx, patient-specific CDSS alerts prompt and guide clinicians to use genetic information when prescribing drugs with known genetically-determined ADRs (Overby et al., 2014; Dunnenberger et al., 2015).

PGx may be implemented either reactively on a gene-by-gene basis at the time of prescribing a drug, or pre-emptively where a single sample is assessed for several pharmacogenes simultaneously with the results stored for future prescribing encounters. Reactive implementation is expensive and has a slow turnaround time and is unsuitable in situations where rapid drug initiation is required. In contrast, pre-emptive screening of multiple pharmacogenes is likely to be more cost-effective and provides the patient with a lifetime’s worth of test results readily available whenever a drug is prescribed, especially when integrated into electronic health records (EHRs) and drug prescription systems (Relling and Evans, 2015). This further underlines the importance of efficient interoperability between different healthcare systems. A patient may be screened for *CYP2C19* prior to being prescribed clopidogrel. These results could inform the prescription of an SSRI or proton pump inhibitor in the future – but only if the results have been stored in an EHR in an accessible format and trigger a CDSS alert at the point of prescription.

### Health informatics

PGx implementation in healthcare can be developed in-house if there is availability of capabilities in laboratory and informatics infrastructure and expertise or outsourced to commercial partners. Due to the considerable diversity in commercial PGx products, it is essential to ensure the clinical, IT integration and interoperability requirements along with robust and continuous updating of evidence are rigorously assessed before deciding on the PGx service provider. The significant costs associated with the use of PGx in clinical practice are now in the domain of decision support, IT integration and interoperability, rather than in laboratory genetic testing (Dunnenberger et al., 2015; Relling and Evans, 2015; van der Wouden et al., 2017). Informatics builds within the EHR are easier for a single gene–drug pair as opposed to the multiple pairs and networks that form as drug interactions and clinical factors are also considered. However, the cost-effectiveness data on the pre-emptive panel approach must be assessed, particularly when considering implementation early in life.

### Patient and provider acceptability

Patient and healthcare professional acceptability is critical for effective and successful PGx implementation. This requires early and continuous engagement with both clinicians and patients, preferably with champions who are committed (Dressler et al., 2018; McDermott et al., 2022). The main barriers to be tackled in the route to implementation are demonstrating that the system will not overburden the physicians, seamlessly integrate into hospital cornerstone systems, provide sufficient support for the users of the system to navigate the pharmacogenetic evidence base through education and decision support systems, demonstrate utility and cost-effectiveness (Stanek et al., 2012; Just et al., 2019; Bagautdinova et al., 2022; Scheuner et al., 2023).

Pharmacists are crucial in the PGx service for evaluating appropriate patient eligibility, providing informative post-test counselling, or leading a PGx consult service (Crews et al., 2011; Brown et al., 2021; Bagautdinova et al., 2022; Krause and Dowd, 2022).

### Health economics

Implementation of PGx in clinical practice requires demonstration of its value and cost-effectiveness to key decision makers and a lack of RCTs that compare genotype-guided prescribing with conventional therapy has not helped. Conducting RCTs for each single drug–gene pair across different ethnicities is not a viable option. Big data analysis of EHRs has the advantage of being able to study diverse populations, limiting concerns about external validity of data and health equality (as is exemplified by the warfarin dosing algorithms that fail to serve patients of African descent). There is limited data on cost-effectiveness multiplexed pre-emptive strategies which are likely to be the preferred solution and the majority of existing cost-effectiveness PGx data are from single gene–drug pair studies (Roden et al., 2018). Most of the cost-effectiveness studies have been conducted separate from implementation initiatives and they indicate that PGx testing results in a reduction in per-patient treatment cost (Winner et al., 2015; Deenen et al., 2016), lower cost-per-QALY (Mitropoulou et al., 2015) and cost savings in long-term care (Saldivar et al., 2016). A recent systematic appraisal of economic evaluations of PGx testing to prevent ADRs found a number of deficiencies in the quality of data used in cost-effectiveness and cost-utility analyses (Turongkaravee et al., 2021). Of the 14 economic evaluation studies of *CYP2C9* and *VKORC1* testing, 10 studies showed that *CYP2C9* and *VKORC1* testing would be a variably cost-effective and four studies suggested otherwise (Turongkaravee et al., 2021). In contrast, all nine economic evaluation studies of *CYP2C19* testing before prescription of clopidogrel ACS patients undergoing PCI showed that *CYP2C19* testing would be a potentially cost-effective treatment strategy for avoiding MACE.

The clopidogrel–*CYP2C19* implementation successes need to be contrasted with the difficulties faced in the implementation of warfarin–*CYP2C9/CYP4F2/VKORC1* PGx. The key enablers for clopidogrel–*CYP2C19* implementation include a discrete patient population (post-PCI), single-gene testing, a high frequency of actionable results, clinically well-established alternative therapies, and a focused group of providers (interventional cardiologists) (Crisamore et al., 2019).

### Implementation in diverse health care systems

Whilst the above discussion related to healthcare systems in high-income countries, the specific challenges in implementing PGx low- and middle-income countries need to be recognised – lack of clinical efficacy and effectiveness data, under-resourced clinical settings, socio-cultural issues and the identification of population specific pharmacogenomic markers (Tata et al., 2020; Magavern et al., 2022; Sukri et al., 2022). The lack of consistent and widely accepted definitions of race, ethnicity and ancestry in genomics and clinical research has resulted in erroneous, inconclusive or absent data on non-European ancestry populations (Popejoy et al., 2020). Initiatives such as Human Heredity and Health in Africa (H3Africa) Consortium and the African Pharmacogenomics Research Consortium attempt to increase the representativeness of pharmacogenomic panels (Matimba et al., 2016). It is imperative that progress in pharmacogenomic research and implementation occurs at pace in diverse populations so that health disparities are not amplified when PGx becomes more mainstream in clinical practice.

#### Author contribution

S.P., C.T. and A.F.D. made substantial contributions to the conception drafting, and revision of the manuscript, it critically for important intellectual content. S.P., C.T. and A.F.D. provided final approval of the version to be published.

## Abbreviations

ABCB1: ATP-binding cassette sub-family B member 1
ACC: American College of Cardiology
ACS: acute coronary syndrome
ADR: adverse drug reaction
ADRB1: β1-adrenergic receptor
ADRB2: β2-adrenergic receptor
AHA: American Heart Association
BMI: body mass index
CDSS: clinical decision support systems
CES1: liver carboxylesterase 1
CPIC: the Clinical Pharmacogenetics Implementation Consortium
CVD: cardiovascular disease
CYP: cytochrome P450
DOAC: direct-acting oral anticoagulants
DPYD: dihydropyrimidine dehydrogenase
DPWG: Dutch Pharmacogenetics Working Groups
EHR: electronic health records
EMA: European Medicines Agency
EPHA7: ephrin type A receptor 7
ESC: European Society of Cardiology
FDA: U.S. Food and Drug Administration
GATM: Glycine amidinotransferase, mitochondrial
GIST: genotype-informed statin therapy
GRK5: G protein-coupled receptor kinase 5
GWAS: genome-wide association studies
HLA-B: human leukocyte antigen B
IM: intermediate metaboliser
INR: international normalised ratio
KCNH2/hERG: human Ether-à-go-go-related gene
LQTS: long QT syndrome
MACE: major adverse cardiovascular event
NAT2: *N*-acetyltransferase 2
NM: normal metaboliser
OATP1B1: organic anion transporting polypeptide 1B1
PCI: percutaneous coronary intervention
PM: poor metaboliser
PGx: pharmacogenomics
QALY: quality-adjusted life year
RCT: randomised controlled trial
SAMS: statin-associated muscle symptoms
SLCO1A2: organic anion transporter family member 1A2
SLCO1B1: solute carrier anion transporter family 1B1
SNP: single nucleotide polymorphism
SSRI: selective serotonin reuptake inhibitors
STEMI: ST-segment elevation myocardial infarction
TdP: torsades des pointes
TIA: transient ischaemic attack
TTR: time in the therapeutic INR range
UGT1A: UDP-glucuronosyltransferase 1A1
UM: ultrarapid metaboliser
VKORC1: vitamin K epoxide reductase complex subunit 1

## References

[r1] Alfirevic A , Neely D , Armitage J , Chinoy H , Cooper RG , Laaksonen R , Carr DF , Bloch KM , Fahy J , Hanson A , Yue QY , Wadelius M , Maitland-van Der Zee AH , Voora D , Psaty BM , Palmer CN and Pirmohamed M (2014) Phenotype standardization for statin-induced myotoxicity. Clinical Pharmacology and Therapeutics 96(4), 470–476. 24897241 10.1038/clpt.2014.121 PMC4172546

[r2] Amstutz U , Henricks LM , Offer SM , Barbarino J , Schellens JHM , Swen JJ , Klein TE , McLeod HL , Caudle KE , Diasio RB and Schwab M (2018) Clinical Pharmacogenetics Implementation Consortium (CPIC) guideline for dihydropyrimidine dehydrogenase genotype and fluoropyrimidine dosing: 2017 update. Clinical Pharmacology and Therapeutics 103(2), 210–216. 29152729 10.1002/cpt.911 PMC5760397

[r3] Angiolillo DJ , Rollini F , Storey RF , Bhatt DL , James S , Schneider DJ , Sibbing D , So DYF , Trenk D , Alexopoulos D , Gurbel PA , Hochholzer W , De Luca L , Bonello L , Aradi D , Cuisset T , Tantry US , Wang TY , Valgimigli M , Waksman R , Mehran R , Montalescot G , Franchi F and Price MJ (2017) International expert consensus on switching platelet P2Y12 receptor-inhibiting therapies. Circulation 136(20), 1955–1975. 29084738 10.1161/CIRCULATIONAHA.117.031164

[r4] Anstensrud AK , Molden E , Haug HJ , Qazi R , Muriq H , Fosshaug LE , Spigset O and Oie E (2020) Impact of genotype-predicted CYP2D6 metabolism on clinical effects and tolerability of metoprolol in patients after myocardial infarction – A prospective observational study. European Journal of Clinical Pharmacology 76(5), 673–683. 31940084 10.1007/s00228-020-02832-0

[r5] Arking DE , Pulit SL , Crotti L , van der Harst P , Munroe PB , Koopmann TT , Sotoodehnia N , Rossin EJ , Morley M , Wang X , Johnson AD , Lundby A , Gudbjartsson DF , Noseworthy PA , Eijgelsheim M , Bradford Y , Tarasov KV , Dorr M , Muller-Nurasyid M , Lahtinen AM , et al. (2014) Genetic association study of QT interval highlights role for calcium signaling pathways in myocardial repolarization. Nature Genetics 46(8), 826–836. 24952745 10.1038/ng.3014 PMC4124521

[r6] Arwood MJ , Deng J , Drozda K , Pugach O , Nutescu EA , Schmidt S , Duarte JD and Cavallari LH (2017) Anticoagulation endpoints with clinical implementation of warfarin pharmacogenetic dosing in a real-world setting: A proposal for a new pharmacogenetic dosing approach. Clinical Pharmacology and Therapeutics 101(5), 675–683. 28032893 10.1002/cpt.558 PMC5395307

[r7] Bagautdinova D , Lteif C , Eddy E , Terrell J , Fisher CL and Duarte JD (2022) Patients’ perspectives of factors that influence pharmacogenetic testing uptake: Enhancing patient counseling and results dissemination. Journal of Personalized Medicine 12(12), 2046. 36556266 10.3390/jpm12122046 PMC9786315

[r8] Bank PCD, Swen JJ and Guchelaar HJ (2019) Estimated nationwide impact of implementing a preemptive pharmacogenetic panel approach to guide drug prescribing in primary care in the Netherlands. BMC Medicine 17(1), 110. 31196067 10.1186/s12916-019-1342-5 PMC6567386

[r9] Batty JA , Hall AS , White HL , Wikstrand J , de Boer RA , van Veldhuisen DJ , van der Harst P , Waagstein F , Hjalmarson A , Kjekshus J , Balmforth AJ and Group M-HS (2014) An investigation of CYP2D6 genotype and response to metoprolol CR/XL during dose titration in patients with heart failure: A MERIT-HF substudy. Clinical Pharmacology and Therapeutics 95(3), 321–330. 24193112 10.1038/clpt.2013.193

[r10] Baudhuin LM , Miller WL , Train L , Bryant S , Hartman KA , Phelps M , Larock M and Jaffe AS (2010) Relation of ADRB1, CYP2D6, and UGT1A1 polymorphisms with dose of, and response to, carvedilol or metoprolol therapy in patients with chronic heart failure. American Journal of Cardiology 106(3), 402–408. 20643254 10.1016/j.amjcard.2010.03.041

[r11] Bauer T , Bouman HJ , van Werkum JW , Ford NF , ten Berg JM and Taubert D (2011) Impact of CYP2C19 variant genotypes on clinical efficacy of antiplatelet treatment with clopidogrel: Systematic review and meta-analysis. BMJ 343, d4588. 21816733 10.1136/bmj.d4588 PMC3191560

[r12] Becker RC , Bassand JP , Budaj A , Wojdyla DM , James SK , Cornel JH , French J , Held C , Horrow J , Husted S , Lopez-Sendon J , Lassila R , Mahaffey KW , Storey RF , Harrington RA and Wallentin L (2011) Bleeding complications with the P2Y12 receptor antagonists clopidogrel and ticagrelor in the PLATelet inhibition and patient outcomes (PLATO) trial. European Heart Journal 32(23), 2933–2944. 22090660 10.1093/eurheartj/ehr422

[r13] Bell GC , Caudle KE , Whirl-Carrillo M , Gordon RJ , Hikino K , Prows CA , Gaedigk A , Agundez J , Sadhasivam S , Klein TE and Schwab M (2017) Clinical Pharmacogenetics Implementation Consortium (CPIC) guideline for CYP2D6 genotype and use of ondansetron and tropisetron. Clinical Pharmacology and Therapeutics 102(2), 213–218. 28002639 10.1002/cpt.598 PMC5479760

[r14] Bijl MJ , Visser LE , van Schaik RH , Kors JA , Witteman JC , Hofman A , Vulto AG , van Gelder T and Stricker BH (2009) Genetic variation in the CYP2D6 gene is associated with a lower heart rate and blood pressure in beta-blocker users. Clinical Pharmacology and Therapeutics 85(1), 45–50. 18784654 10.1038/clpt.2008.172

[r15] Blake CM , Kharasch ED , Schwab M and Nagele P (2013) A meta-analysis of CYP2D6 metabolizer phenotype and metoprolol pharmacokinetics. Clinical Pharmacology and Therapeutics 94(3), 394–399. 23665868 10.1038/clpt.2013.96 PMC3818912

[r16] Bourgeois S , Jorgensen A , Zhang EJ , Hanson A , Gillman MS , Bumpstead S , Toh CH , Williamson P , Daly AK , Kamali F , Deloukas P and Pirmohamed M (2016) A multi-factorial analysis of response to warfarin in a UK prospective cohort. Genome Medicine 8(1), 2. 26739746 10.1186/s13073-015-0255-y PMC4702374

[r17] Brouwer J , Nijenhuis M , Soree B , Guchelaar HJ , Swen JJ , van Schaik RHN , Weide JV , Rongen G , Buunk AM , de Boer-Veger NJ , Houwink EJF , van Westrhenen R , Wilffert B , Deneer VHM and Mulder H (2022) Dutch Pharmacogenetics Working Group (DPWG) guideline for the gene-drug interaction between CYP2C19 and CYP2D6 and SSRIs. European Journal of Human Genetics 30, 1114–1120. 34782755 10.1038/s41431-021-01004-7 PMC9553948

[r18] Brown JT , MacDonald D , Yapel A , Luczak T , Hanson A and Stenehjem DD (2021) Integrating pharmacogenetic testing via medication therapy management in an outpatient family medicine clinic. Pharmacogenomics 22(4), 203–212. 33470873 10.2217/pgs-2020-0178

[r19] Cancino RS , Hylek EM , Reisman JI and Rose AJ (2014) Comparing patient-level and site-level anticoagulation control as predictors of adverse events. Thrombosis Research 133(4), 652–656. 24502961 10.1016/j.thromres.2014.01.013

[r20] Carr DF , Francis B , Jorgensen AL , Zhang E , Chinoy H , Heckbert SR , Bis JC , Brody JA , Floyd JS , Psaty BM , Molokhia M , Lapeyre-Mestre M , Conforti A , Alfirevic A , van Staa T and Pirmohamed M (2019) Genomewide association study of statin-induced myopathy in patients recruited using the UK clinical practice research datalink. Clinical Pharmacology and Therapeutics 106(6), 1353–1361. 31220337 10.1002/cpt.1557 PMC6896237

[r21] Carreras ET , Hochholzer W , Frelinger AL , Nordio F , O’Donoghue ML , Wiviott SD , Angiolillo DJ , Michelson AD , Sabatine MS and Mega JL (2016) Diabetes mellitus, CYP2C19 genotype, and response to escalating doses of clopidogrel. Insights from the ELEVATE-TIMI 56 trial. Thrombosis and Haemostasis 116(1), 69–77. 27009617 10.1160/TH15-12-0981

[r22] Catapano AL , Graham I , De Backer G , Wiklund O , Chapman MJ , Drexel H , Hoes AW , Jennings CS , Landmesser U , Pedersen TR , Reiner Z , Riccardi G , Taskinen MR , Tokgozoglu L , Verschuren WMM , Vlachopoulos C , Wood DA , Zamorano JL , Cooney MT and Group ESCSD (2016) 2016 ESC/EAS guidelines for the management of dyslipidaemias. European Heart Journal 37(39), 2999–3058. 27567407 10.1093/eurheartj/ehw272

[r23] Cavallari LH , Lee CR , Beitelshees AL , Cooper-DeHoff RM , Duarte JD , Voora D , Kimmel SE , McDonough CW , Gong Y , Dave CV , Pratt VM , Alestock TD , Anderson RD , Alsip J , Ardati AK , Brott BC , Brown L , Chumnumwat S , Clare-Salzler MJ , Coons JC , Denny JC , Dillon C , Elsey AR , Hamadeh IS , Harada S , Hillegass WB , Hines L , Horenstein RB , Howell LA , Jeng LJB , Kelemen MD , Lee YM , Magvanjav O , Montasser M , Nelson DR , Nutescu EA , Nwaba DC , Pakyz RE , Palmer K , Peterson JF , Pollin TI , Quinn AH , Robinson SW , Schub J , Skaar TC , Smith DM , Sriramoju VB , Starostik P , Stys TP , Stevenson JM , Varunok N , Vesely MR , Wake DT , Weck KE , Weitzel KW , Wilke RA , Willig J , Zhao RY , Kreutz RP , Stouffer GA , Empey PE , Limdi NA , Shuldiner AR , Winterstein AG , Johnson JA and Network I (2018) Multisite investigation of outcomes with implementation of CYP2C19 genotype-guided antiplatelet therapy after percutaneous coronary intervention. JACC. Cardiovascular Interventions 11(2), 181–191. 29102571 10.1016/j.jcin.2017.07.022 PMC5775044

[r24] Chanfreau-Coffinier C , Hull LE , Lynch JA , DuVall SL , Damrauer SM , Cunningham FE , Voight BF , Matheny ME , Oslin DW , Icardi MS and Tuteja S (2019) Projected prevalence of actionable pharmacogenetic variants and level A drugs prescribed among US veterans health administration pharmacy users. JAMA Network Open 2(6), e195345. 31173123 10.1001/jamanetworkopen.2019.5345 PMC6563578

[r25] Choi HY , Bae KS , Cho SH , Ghim JL , Choe S , Jung JA , Jin SJ , Kim HS and Lim HS (2015) Impact of CYP2D6, CYP3A5, CYP2C19, CYP2A6, SLCO1B1, ABCB1, and ABCG2 gene polymorphisms on the pharmacokinetics of simvastatin and simvastatin acid. Pharmacogenetics and Genomics 25(12), 595–608. 26367500 10.1097/FPC.0000000000000176

[r26] Claassens DM and Sibbing D (2020) De-escalation of antiplatelet treatment in patients with myocardial infarction who underwent percutaneous coronary intervention: A review of the current literature. Journal of Clinical Medicine 9(9), 2983. 32942754 10.3390/jcm9092983 PMC7563354

[r27] Claassens DMF , Vos GJA , Bergmeijer TO, Hermanides RS , van’t Hof AWJ , van der Harst P , Barbato E , Morisco C , Tjon Joe Gin RM , Asselbergs FW , Mosterd A , Herrman JR , Dewilde WJM , Janssen PWA , Kelder JC , Postma MJ , de Boer A , Boersma C , Deneer VHM and Ten Berg JM (2019) A genotype-guided strategy for oral P2Y12 inhibitors in primary PCI. New England Journal of Medicine 381(17), 1621–1631. 31479209 10.1056/NEJMoa1907096

[r28] Collet JP , Thiele H , Barbato E , Barthelemy O , Bauersachs J , Bhatt DL , Dendale P , Dorobantu M , Edvardsen T , Folliguet T , Gale CP , Gilard M , Jobs A , Juni P , Lambrinou E , Lewis BS , Mehilli J , Meliga E , Merkely B , Mueller C , Roffi M , Rutten FH , Sibbing D , Siontis GCM and Group ESCSD (2021) 2020 ESC guidelines for the management of acute coronary syndromes in patients presenting without persistent ST-segment elevation. European Heart Journal 42(14), 1289–1367. 32860058 10.1093/eurheartj/ehaa575

[r29] Collins FS and Varmus H (2015) A new initiative on precision medicine. New England Journal of Medicine 372(9), 793–795. 25635347 10.1056/NEJMp1500523 PMC5101938

[r30] Cooper GM , Johnson JA , Langaee TY , Feng H , Stanaway IB , Schwarz UI , Ritchie MD , Stein CM , Roden DM , Smith JD , Veenstra DL , Rettie AE and Rieder MJ (2008) A genome-wide scan for common genetic variants with a large influence on warfarin maintenance dose. Blood 112(4), 1022–1027. 18535201 10.1182/blood-2008-01-134247 PMC2515139

[r31] CPIC (2022) Genes-drugs. Available at https://cpicpgx.org/genes-drugs/ (accessed 20 March 2023).

[r32] Crews KR , Cross SJ , McCormick JN , Baker DK , Molinelli AR , Mullins R , Relling MV and Hoffman JM (2011) Development and implementation of a pharmacist-managed clinical pharmacogenetics service. American Journal of Health-System Pharmacy 68(2), 143–150. 21200062 10.2146/ajhp100113 PMC3228517

[r33] Crews KR , Gaedigk A , Dunnenberger HM , Leeder JS , Klein TE , Caudle KE , Haidar CE , Shen DD , Callaghan JT , Sadhasivam S , Prows CA , Kharasch ED , Skaar TC and Clinical Pharmacogenetics Implementation (2014) Clinical Pharmacogenetics Implementation Consortium guidelines for cytochrome P450 2D6 genotype and codeine therapy: 2014 update. Clinical Pharmacology and Therapeutics 95(4), 376–382. 24458010 10.1038/clpt.2013.254 PMC3975212

[r34] Crisamore KR , Nolin TD , Coons JC and Empey PE (2019) Engaging and empowering stakeholders to advance pharmacogenomics. Clinical Pharmacology and Therapeutics 106(2), 305–308. 31241758 10.1002/cpt.1470

[r35] Danese E , Raimondi S , Montagnana M , Tagetti A , Langaee T , Borgiani P , Ciccacci C , Carcas AJ , Borobia AM , Tong HY , Davila-Fajardo C , Rodrigues Botton M , Bourgeois S , Deloukas P , Caldwell MD , Burmester JK , Berg RL , Cavallari LH , Drozda K , Huang M , Zhao LZ , Cen HJ , Gonzalez-Conejero R , Roldan V , Nakamura Y , Mushiroda T , Gong IY , Kim RB , Hirai K , Itoh K , Isaza C , Beltran L , Jimenez-Varo E , Canadas-Garre M , Giontella A , Kringen MK , Haug KBF , Gwak HS , Lee KE , Minuz P , Lee MTM , Lubitz SA , Scott S , Mazzaccara C , Sacchetti L , Genc E , Ozer M , Pathare A , Krishnamoorthy R , Paldi A , Siguret V , Loriot MA , Kutala VK , Suarez-Kurtz G , Perini J , Denny JC , Ramirez AH , Mittal B , Rathore SS , Sagreiya H , Altman R , Shahin MHA , Khalifa SI , Limdi NA , Rivers C , Shendre A , Dillon C , Suriapranata IM , Zhou HH , Tan SL , Tatarunas V , Lesauskaite V , Zhang Y , Maitland-van der Zee AH , Verhoef TI , de Boer A , Taljaard M , Zambon CF , Pengo V , Zhang JE , Pirmohamed M , Johnson JA and Fava C (2019) Effect of CYP4F2, VKORC1, and CYP2C9 in influencing coumarin dose: A single-patient data meta-analysis in more than 15,000 individuals. Clinical Pharmacology and Therapeutics 105(6), 1477–1491. 30506689 10.1002/cpt.1323 PMC6542461

[r36] Danik JS , Chasman DI , MacFadyen JG , Nyberg F , Barratt BJ and Ridker PM (2013) Lack of association between SLCO1B1 polymorphisms and clinical myalgia following rosuvastatin therapy. American Heart Journal 165(6), 1008–1014. 23708174 10.1016/j.ahj.2013.01.025

[r37] de Keyser CE , Peters BJ , Becker ML , Visser LE , Uitterlinden AG , Klungel OH , Verstuyft C , Hofman A , Maitland-van der Zee AH and Stricker BH (2014) The SLCO1B1 c.521T>C polymorphism is associated with dose decrease or switching during statin therapy in the Rotterdam study. Pharmacogenetics and Genomics 24(1), 43–51. 24263182 10.1097/FPC.0000000000000018

[r38] De T , Alarcon C , Hernandez W , Liko I , Cavallari LH , Duarte JD and Perera MA (2018) Association of genetic variants with warfarin-associated bleeding among patients of African descent. JAMA 320(16), 1670–1677. 30357299 10.1001/jama.2018.14955 PMC6233811

[r39] Deenen MJ , Meulendijks D , Cats A , Sechterberger MK , Severens JL , Boot H , Smits PH , Rosing H , Mandigers CM , Soesan M , Beijnen JH and Schellens JH (2016) Upfront genotyping of DPYD*2A to individualize fluoropyrimidine therapy: A safety and cost analysis. Journal of Clinical Oncology 34(3), 227–234. 26573078 10.1200/JCO.2015.63.1325

[r40] Diaz-Villamarin X , Davila-Fajardo CL , Martinez-Gonzalez LJ , Carmona-Saez P , Sanchez-Ramos J , Alvarez Cubero MJ , Salmeron-Febres LM , Cabeza Barrera J and Fernandez-Quesada F (2016) Genetic polymorphisms influence on the response to clopidogrel in peripheral artery disease patients following percutaneous transluminal angioplasty. Pharmacogenomics 17(12), 1327–1338. 27464309 10.2217/pgs-2016-0056

[r41] Doki K , Sekiguchi Y , Kuga K , Aonuma K and Homma M (2015) Serum flecainide S/R ratio reflects the CYP2D6 genotype and changes in CYP2D6 activity. Drug Metabolism and Pharmacokinetics 30(4), 257–262. 26195225 10.1016/j.dmpk.2015.04.001

[r42] Dressler LG , Bell GC , Ruch KD , Retamal JD , Krug PB and Paulus RA (2018) Implementing a personalized medicine program in a community health system. Pharmacogenomics 19(17), 1345–1356. 30345883 10.2217/pgs-2018-0130

[r43] Drozda K , Wong S , Patel SR , Bress AP , Nutescu EA , Kittles RA and Cavallari LH (2015) Poor warfarin dose prediction with pharmacogenetic algorithms that exclude genotypes important for African Americans. Pharmacogenetics and Genomics 25(2), 73–81. 25461246 10.1097/FPC.0000000000000108 PMC4280295

[r44] Dunnenberger HM , Crews KR , Hoffman JM , Caudle KE , Broeckel U , Howard SC , Hunkler RJ , Klein TE , Evans WE and Relling MV (2015) Preemptive clinical pharmacogenetics implementation: Current programs in five US medical centers. Annual Review of Pharmacology and Toxicology 55, 89–106. 10.1146/annurev-pharmtox-010814-124835 PMC4607278 25292429

[r45] Ehmann F , Caneva L , Prasad K , Paulmichl M , Maliepaard M , Llerena A , Ingelman-Sundberg M and Papaluca-Amati M (2015) Pharmacogenomic information in drug labels: European medicines agency perspective. Pharmacogenomics Journal 15(3), 201–210. 25707393 10.1038/tpj.2014.86

[r46] Empey PE , Stevenson JM , Tuteja S , Weitzel KW , Angiolillo DJ , Beitelshees AL , Coons JC , Duarte JD , Franchi F , Jeng LJB , Johnson JA , Kreutz RP , Limdi NA , Maloney KA , Owusu Obeng A , Peterson JF , Petry N , Pratt VM , Rollini F , Scott SA , Skaar TC , Vesely MR , Stouffer GA , Wilke RA , Cavallari LH , Lee CR and Network I (2018) Multisite investigation of strategies for the implementation of CYP2C19 genotype-guided antiplatelet therapy. Clinical Pharmacology and Therapeutics 104(4), 664–674. 29280137 10.1002/cpt.1006 PMC6019555

[r47] FDA (2023a) Table of Pharmacogenetic Associations. U.S. Food and Drug Administration. Available at https://www.fda.gov/medical-devices/precision-medicine/table-pharmacogenetic-associations (accessed 20 March 2023).

[r48] FDA (2023b) Table of Pharmacogenomic Biomarkers in Drug Labeling. U.S. Food and Drug Administration. Available at https://www.fda.gov/drugs/science-and-research-drugs/table-pharmacogenomic-biomarkers-drug-labeling (accessed 20 March 2023).

[r49] Fiegenbaum M , da Silveira FR , Van der Sand CR , Van der Sand LC , Ferreira ME , Pires RC and Hutz MH (2005) The role of common variants of ABCB1, CYP3A4, and CYP3A5 genes in lipid-lowering efficacy and safety of simvastatin treatment. Clinical Pharmacology and Therapeutics 78(5), 551–558. 16321621 10.1016/j.clpt.2005.08.003

[r50] Gage BF , Bass AR , Lin H , Woller SC , Stevens SM , Al-Hammadi N , Li J , Rodriguez T , Miller JP , McMillin GA , Pendleton RC , Jaffer AK , King CR , Whipple BD , Porche-Sorbet R , Napoli L , Merritt K , Thompson AM , Hyun G , Anderson JL , Hollomon W , Barrack RL , Nunley RM , Moskowitz G , Davila-Roman V and Eby CS (2017) Effect of genotype-guided warfarin dosing on clinical events and anticoagulation control among patients undergoing hip or knee arthroplasty: The GIFT randomized clinical trial. JAMA 318(12), 1115–1124. 28973620 10.1001/jama.2017.11469 PMC5818817

[r51] Gage BF , Eby C , Johnson JA , Deych E , Rieder MJ , Ridker PM , Milligan PE , Grice G , Lenzini P , Rettie AE , Aquilante CL , Grosso L , Marsh S , Langaee T , Farnett LE , Voora D , Veenstra DL , Glynn RJ , Barrett A and McLeod HL (2008) Use of pharmacogenetic and clinical factors to predict the therapeutic dose of warfarin. Clinical Pharmacology and Therapeutics 84(3), 326–331. 18305455 10.1038/clpt.2008.10 PMC2683977

[r52] Goldberger JJ and Buxton AE (2013) Personalized medicine vs guideline-based medicine. JAMA 309(24), 2559–2560. 23712449 10.1001/jama.2013.6629

[r53] Gonzalez-Fierro A , Vasquez-Bahena D , Taja-Chayeb L , Vidal S , Trejo-Becerril C , Perez-Cardenas E , de la Cruz-Hernandez E , Chavez-Blanco A , Gutierrez O , Rodriguez D , Fernandez Z and Duenas-Gonzalez A (2011) Pharmacokinetics of hydralazine, an antihypertensive and DNA-demethylating agent, using controlled-release formulations designed for use in dosing schedules based on the acetylator phenotype. International Journal of Clinical Pharmacology and Therapeutics 49(8), 519–524. 21781652 10.5414/cp201526

[r54] Greden JF , Parikh SV , Rothschild AJ , Thase ME , Dunlop BW , DeBattista C , Conway CR , Forester BP , Mondimore FM , Shelton RC , Macaluso M , Li J , Brown K , Gilbert A , Burns L , Jablonski MR and Dechairo B (2019) Impact of pharmacogenomics on clinical outcomes in major depressive disorder in the GUIDED trial: A large, patient- and rater-blinded, randomized, controlled study. Journal of Psychiatric Research 111, 59–67. 30677646 10.1016/j.jpsychires.2019.01.003

[r55] Guo B , Tan Q , Guo D , Shi Z , Zhang C and Guo W (2014) Patients carrying CYP2C19 loss of function alleles have a reduced response to clopidogrel therapy and a greater risk of in-stent restenosis after endovascular treatment of lower extremity peripheral arterial disease. Journal of Vascular Surgery 60(4), 993–1001. 24877854 10.1016/j.jvs.2014.03.293

[r56] Hamadeh IS , Langaee TY , Dwivedi R , Garcia S , Burkley BM , Skaar TC , Chapman AB , Gums JG , Turner ST , Gong Y , Cooper-DeHoff RM and Johnson JA (2014) Impact of CYP2D6 polymorphisms on clinical efficacy and tolerability of metoprolol tartrate. Clinical Pharmacology and Therapeutics 96(2), 175–181. 24637943 10.1038/clpt.2014.62 PMC4111800

[r57] Han LW , Ryu RJ , Cusumano M , Easterling TR , Phillips BR , Risler LJ , Shen DD and Hebert MF (2019) Effect of N-acetyltransferase 2 genotype on the pharmacokinetics of hydralazine during pregnancy. Journal of Clinical Pharmacology 59(12), 1678–1689. 31257615 10.1002/jcph.1477 PMC6813860

[r58] Heise CW , Gallo T , Curry SC and Woosley RL (2020) Identification of populations likely to benefit from pharmacogenomic testing. Pharmacogenetics and Genomics 30(5), 91–95. 32209836 10.1097/FPC.0000000000000400

[r59] Hicks JK , Bishop JR , Sangkuhl K , Muller DJ , Ji Y , Leckband SG , Leeder JS , Graham RL , Chiulli DL , LLerena A , Skaar TC , Scott SA , Stingl JC , Klein TE , Caudle KE , Gaedigk A and Clinical Pharmacogenetics Implementation (2015) Clinical Pharmacogenetics Implementation Consortium (CPIC) guideline for CYP2D6 and CYP2C19 genotypes and dosing of selective serotonin reuptake inhibitors. Clinical Pharmacology and Therapeutics 98(2), 127–134. 25974703 10.1002/cpt.147 PMC4512908

[r60] Hicks JK , El Rouby N , Ong HH , Schildcrout JS , Ramsey LB , Shi Y , Anne Tang L , Aquilante CL , Beitelshees AL , Blake KV , Cimino JJ , Davis BH , Empey PE , Kao DP , Lemkin DL , Limdi NA , G PL , Rosenman MB , Skaar TC , Teal E , Tuteja S , Wiley LK , Williams H , Winterstein AG , Van Driest SL , Cavallari LH , Peterson JF and Group IPW (2021) Opportunity for genotype-guided prescribing among adult patients in 11 US health systems. Clinical Pharmacology and Therapeutics 110(1), 179–188. 33428770 10.1002/cpt.2161 PMC8217370

[r61] Ho KH , van Hove M and Leng G (2020) Trends in anticoagulant prescribing: A review of local policies in English primary care. BMC Health Services Research 20(1), 279. 32245380 10.1186/s12913-020-5058-1 PMC7126454

[r62] Hoenig MR , Walker PJ , Gurnsey C , Beadle K and Johnson L (2011) The C3435T polymorphism in ABCB1 influences atorvastatin efficacy and muscle symptoms in a high-risk vascular cohort. Journal of Clinical Lipidology 5(2), 91–96. 21392722 10.1016/j.jacl.2011.01.001

[r63] Holmes DR , Dehmer GJ , Kaul S , Leifer D , O’Gara PT and Stein CM (2010) ACCF/AHA clopidogrel clinical alert: Approaches to the FDA “boxed warning”: A report of the American College of Cardiology Foundation task force on clinical expert consensus documents and the American Heart Association endorsed by the Society for Cardiovascular Angiography and Interventions and the Society of Thoracic Surgeons. Journal of the American College of Cardiology 56(4), 321–341. 20633831 10.1016/j.jacc.2010.05.013

[r64] Holmes MV , Perel P , Shah T , Hingorani AD and Casas JP (2011) CYP2C19 genotype, clopidogrel metabolism, platelet function, and cardiovascular events: A systematic review and meta-analysis. JAMA 306(24), 2704–2714. 22203539 10.1001/jama.2011.1880

[r65] Huang J , Li C , Song Y , Fan X , You L , Tan L , Xiao L , Li Q , Ruan G , Hu S , Cui W , Li Z , Ni L , Chen C , Woo AY , Xiao RP and Wang DW (2018) ADRB2 polymorphism Arg16Gly modifies the natural outcome of heart failure and dictates therapeutic response to beta-blockers in patients with heart failure. Cell Discovery 4, 57. 30374408 10.1038/s41421-018-0058-6 PMC6198009

[r66] Hulot JS , Collet JP , Silvain J , Pena A , Bellemain-Appaix A , Barthelemy O , Cayla G , Beygui F and Montalescot G (2010) Cardiovascular risk in clopidogrel-treated patients according to cytochrome P450 2C19*2 loss-of-function allele or proton pump inhibitor coadministration: A systematic meta-analysis. Journal of the American College of Cardiology 56(2), 134–143. 20620727 10.1016/j.jacc.2009.12.071

[r67] Hylek EM , Evans-Molina C , Shea C , Henault LE and Regan S (2007) Major hemorrhage and tolerability of warfarin in the first year of therapy among elderly patients with atrial fibrillation. Circulation 115(21), 2689–2696. 17515465 10.1161/CIRCULATIONAHA.106.653048

[r68] Ibanez B , James S , Agewall S , Antunes MJ , Bucciarelli-Ducci C , Bueno H , Caforio ALP , Crea F , Goudevenos JA , Halvorsen S , Hindricks G , Kastrati A , Lenzen MJ , Prescott E , Roffi M , Valgimigli M , Varenhorst C , Vranckx P , Widimsky P and Group ESCSD (2018) 2017 ESC guidelines for the management of acute myocardial infarction in patients presenting with ST-segment elevation: The task force for the management of acute myocardial infarction in patients presenting with ST-segment elevation of the European Society of Cardiology (ESC). European Heart Journal 39(2), 119–177.28886621

[r69] Ingelman-Sundberg M , Sim SC , Gomez A and Rodriguez-Antona C (2007) Influence of cytochrome P450 polymorphisms on drug therapies: Pharmacogenetic, pharmacoepigenetic and clinical aspects. Pharmacology & Therapeutics 116(3), 496–526. 18001838 10.1016/j.pharmthera.2007.09.004

[r70] International Warfarin Pharmacogenetics, Klein TE , Altman RB , Eriksson N , Gage BF , Kimmel SE , Lee MT , Limdi NA , Page D , Roden DM , Wagner MJ , Caldwell MD and Johnson JA (2009) Estimation of the warfarin dose with clinical and pharmacogenetic data. New England Journal of Medicine 360(8), 753–764. 19228618 10.1056/NEJMoa0809329 PMC2722908

[r71] Johnson JA , Caudle KE , Gong L , Whirl-Carrillo M , Stein CM , Scott SA , Lee MT , Gage BF , Kimmel SE , Perera MA , Anderson JL , Pirmohamed M , Klein TE , Limdi NA , Cavallari LH and Wadelius M (2017) Clinical Pharmacogenetics Implementation Consortium (CPIC) guideline for pharmacogenetics-guided warfarin dosing: 2017 update. Clinical Pharmacology and Therapeutics 102(3), 397–404. 28198005 10.1002/cpt.668 PMC5546947

[r72] Jones M , McEwan P , Morgan CL , Peters JR , Goodfellow J and Currie CJ (2005) Evaluation of the pattern of treatment, level of anticoagulation control, and outcome of treatment with warfarin in patients with non-valvar atrial fibrillation: A record linkage study in a large British population. Heart 91(4), 472–477. 15772203 10.1136/hrt.2004.042465 PMC1768813

[r73] Just KS , Turner RM , Dolzan V , Cecchin E , Swen JJ , Gurwitz D and Stingl JC (2019) Educating the next generation of pharmacogenomics experts: Global educational needs and concepts. Clinical Pharmacology and Therapeutics 106(2), 313–316. 31237679 10.1002/cpt.1471 PMC6771464

[r74] Kaminsky LS and Zhang ZY (1997) Human P450 metabolism of warfarin. Pharmacology & Therapeutics 73(1), 67–74. 9014207 10.1016/s0163-7258(96)00140-4

[r75] Kazui M , Nishiya Y , Ishizuka T , Hagihara K , Farid NA , Okazaki O , Ikeda T and Kurihara A (2010) Identification of the human cytochrome P450 enzymes involved in the two oxidative steps in the bioactivation of clopidogrel to its pharmacologically active metabolite. Drug Metabolism and Disposition 38(1), 92–99. 19812348 10.1124/dmd.109.029132

[r76] Kheiri B , Simpson TF , Osman M , Kumar K , Przybylowicz R , Merrill M , Golwala H , Rahmouni H , Cigarroa JE and Zahr F (2020) Genotype-guided strategy for P2Y12 inhibitors in coronary artery disease: A meta-analysis of randomized clinical trials. JACC. Cardiovascular Interventions 13(5), 659–661. 32139230 10.1016/j.jcin.2019.11.019

[r77] Kimmel SE , French B , Kasner SE , Johnson JA , Anderson JL , Gage BF , Rosenberg YD , Eby CS , Madigan RA , McBane RB , Abdel-Rahman SZ , Stevens SM , Yale S , Mohler ER, III, Fang MC , Shah V , Horenstein RB , Limdi NA , Muldowney JA, III, Gujral J , Delafontaine P , Desnick RJ , Ortel TL , Billett HH , Pendleton RC , Geller NL , Halperin JL , Goldhaber SZ , Caldwell MD , Califf RM , Ellenberg JH and Investigators C (2013) A pharmacogenetic versus a clinical algorithm for warfarin dosing. New England Journal of Medicine 369(24), 2283–2293. 24251361 10.1056/NEJMoa1310669 PMC3942158

[r78] Krause DS and Dowd D (2022) Use of a consultation service following pharmacogenetic testing in psychiatry. Pharmacogenomics 23(5), 327–333. 35296147 10.2217/pgs-2021-0121

[r79] Lamoureux F , Duflot T and French Network of Pharmacogenetics (2017) Pharmacogenetics in cardiovascular diseases: State of the art and implementation-recommendations of the French National Network of pharmacogenetics (RNPGx). Thérapie 72(2), 257–267. 28237404 10.1016/j.therap.2016.09.017

[r80] Landefeld CS and Beyth RJ (1993) Anticoagulant-related bleeding: Clinical epidemiology, prediction, and prevention. American Journal of Medicine 95(3), 315–328. 8368229 10.1016/0002-9343(93)90285-w

[r81] Lau WCY , Li X , Wong ICK , Man KKC , Lip GYH , Leung WK , Siu CW and Chan EW (2017) Bleeding-related hospital admissions and 30-day readmissions in patients with non-valvular atrial fibrillation treated with dabigatran versus warfarin. Journal of Thrombosis and Haemostasis 15(10), 1923–1933. 28748652 10.1111/jth.13780

[r82] Lee CR , Luzum JA , Sangkuhl K , Gammal RS , Sabatine MS , Stein CM , Kisor DF , Limdi NA , Lee YM , Scott SA , Hulot JS , Roden DM , Gaedigk A , Caudle KE , Klein TE , Johnson JA and Shuldiner AR (2022) Clinical Pharmacogenetics Implementation Consortium guideline for CYP2C19 genotype and clopidogrel therapy: 2022 update. Clinical Pharmacology and Therapeutics 112(5), 959–967. 35034351 10.1002/cpt.2526 PMC9287492

[r83] Lim KS , Jang IJ , Kim BH , Kim J , Jeon JY , Tae YM , Yi S , Eum S , Cho JY , Shin SG and Yu KS (2010) Changes in the QTc interval after administration of flecainide acetate, with and without coadministered paroxetine, in relation to cytochrome P450 2D6 genotype: Data from an open-label, two-period, single-sequence crossover study in healthy Korean male subjects. Clinical Therapeutics 32(4), 659–666. 20435235 10.1016/j.clinthera.2010.04.002

[r84] Lima JJ , Thomas CD , Barbarino J , Desta Z , Van Driest SL , El Rouby N , Johnson JA , Cavallari LH , Shakhnovich V , Thacker DL , Scott SA , Schwab M , Uppugunduri CRS , Formea CM , Franciosi JP , Sangkuhl K , Gaedigk A , Klein TE , Gammal RS and Furuta T (2021) Clinical Pharmacogenetics Implementation Consortium (CPIC) guideline for CYP2C19 and proton pump inhibitor dosing. Clinical Pharmacology and Therapeutics 109(6), 1417–1423. 32770672 10.1002/cpt.2015 PMC7868475

[r85] Limdi NA , Wadelius M , Cavallari L , Eriksson N , Crawford DC , Lee MT , Chen CH , Motsinger-Reif A , Sagreiya H , Liu N , Wu AH , Gage BF , Jorgensen A , Pirmohamed M , Shin JG , Suarez-Kurtz G , Kimmel SE , Johnson JA , Klein TE , Wagner MJ and International Warfarin Pharmacogenetics (2010) Warfarin pharmacogenetics: A single VKORC1 polymorphism is predictive of dose across 3 racial groups. Blood 115(18), 3827–3834. 20203262 10.1182/blood-2009-12-255992 PMC2865873

[r86] Linskey DW , English JD , Perry DA , Ochs-Balcom HM , Ma C , Isackson PJ , Vladutiu GD and Luzum JA (2020) Association of SLCO1B1 c.521T>C (rs4149056) with discontinuation of atorvastatin due to statin-associated muscle symptoms. Pharmacogenetics and Genomics 30(9), 208–211. 32453264 10.1097/FPC.0000000000000412 PMC8062056

[r87] Magavern EF , Gurdasani D , Ng FL and Lee SS (2022) Health equality, race and pharmacogenomics. British Journal of Clinical Pharmacology 88(1), 27–33. 34251046 10.1111/bcp.14983 PMC8752640

[r88] Magvanjav O , McDonough CW , Gong Y , McClure LA , Talbert RL , Horenstein RB , Shuldiner AR , Benavente OR , Mitchell BD , Johnson JA and SiGN N (2017) Pharmacogenetic associations of beta1-adrenergic receptor polymorphisms with cardiovascular outcomes in the SPS3 trial (secondary prevention of small subcortical strokes). Stroke 48(5), 1337–1343. 28351962 10.1161/STROKEAHA.116.015936 PMC5404951

[r89] Mallal S , Phillips E , Carosi G , Molina JM , Workman C , Tomazic J , Jagel-Guedes E , Rugina S , Kozyrev O , Cid JF , Hay P , Nolan D , Hughes S , Hughes A , Ryan S , Fitch N , Thorborn D , Benbow A and Team P-S (2008) HLA-B*5701 screening for hypersensitivity to abacavir. New England Journal of Medicine 358(6), 568–579. 18256392 10.1056/NEJMoa0706135

[r90] Mangravite LM , Engelhardt BE , Medina MW , Smith JD , Brown CD , Chasman DI , Mecham BH , Howie B , Shim H , Naidoo D , Feng Q , Rieder MJ , Chen YD , Rotter JI , Ridker PM , Hopewell JC , Parish S , Armitage J , Collins R , Wilke RA , Nickerson DA , Stephens M and Krauss RM (2013) A statin-dependent QTL for GATM expression is associated with statin-induced myopathy. Nature 502(7471), 377–380. 23995691 10.1038/nature12508 PMC3933266

[r91] Manolio TA , Chisholm RL , Ozenberger B , Roden DM , Williams MS , Wilson R , Bick D , Bottinger EP , Brilliant MH , Eng C , Frazer KA , Korf B , Ledbetter DH , Lupski JR , Marsh C , Mrazek D , Murray MF , O’Donnell PH , Rader DJ , Relling MV , Shuldiner AR , Valle D , Weinshilboum R , Green ED and Ginsburg GS (2013) Implementing genomic medicine in the clinic: The future is here. Genetics in Medicine 15(4), 258–267. 23306799 10.1038/gim.2012.157 PMC3835144

[r92] Matimba A , Dhoro M and Dandara C (2016) Is there a role of pharmacogenomics in Africa. Global Health, Epidemiology and Genomics 1, e9. 29868201 10.1017/gheg.2016.4 PMC5870419

[r93] Mazari L , Ouarzane M and Zouali M (2007) Subversion of B lymphocyte tolerance by hydralazine, a potential mechanism for drug-induced lupus. Proceedings of the National Academy of Sciences of the United States of America 104(15), 6317–6322. 17404230 10.1073/pnas.0610434104 PMC1851062

[r94] McDermott JH , Wright S , Sharma V , Newman WG , Payne K and Wilson P (2022) Characterizing pharmacogenetic programs using the consolidated framework for implementation research: A structured scoping review. Frontiers in Medicine (Lausanne) 9, 945352. 10.3389/fmed.2022.945352 PMC9433561 36059837

[r95] Mega JL , Close SL , Wiviott SD , Shen L , Hockett RD , Brandt JT , Walker JR , Antman EM , Macias W , Braunwald E and Sabatine MS (2009a) Cytochrome p-450 polymorphisms and response to clopidogrel. New England Journal of Medicine 360(4), 354–362. 19106084 10.1056/NEJMoa0809171

[r96] Mega JL , Close SL , Wiviott SD , Shen L , Hockett RD , Brandt JT , Walker JR , Antman EM , Macias WL , Braunwald E and Sabatine MS (2009b) Cytochrome P450 genetic polymorphisms and the response to prasugrel: Relationship to pharmacokinetic, pharmacodynamic, and clinical outcomes. Circulation 119(19), 2553–2560. 19414633 10.1161/CIRCULATIONAHA.109.851949

[r97] Mega JL , Hochholzer W , Frelinger AL, III, Kluk MJ , Angiolillo DJ , Kereiakes DJ , Isserman S , Rogers WJ , Ruff CT , Contant C , Pencina MJ , Scirica BM , Longtine JA , Michelson AD and Sabatine MS (2011) Dosing clopidogrel based on CYP2C19 genotype and the effect on platelet reactivity in patients with stable cardiovascular disease. JAMA 306(20), 2221–2228. 22088980 10.1001/jama.2011.1703

[r98] Mega JL , Simon T , Collet JP , Anderson JL , Antman EM , Bliden K , Cannon CP , Danchin N , Giusti B , Gurbel P , Horne BD , Hulot JS , Kastrati A , Montalescot G , Neumann FJ , Shen L , Sibbing D , Steg PG , Trenk D , Wiviott SD and Sabatine MS (2010) Reduced-function CYP2C19 genotype and risk of adverse clinical outcomes among patients treated with clopidogrel predominantly for PCI: A meta-analysis. JAMA 304(16), 1821–1830. 20978260 10.1001/jama.2010.1543 PMC3048820

[r99] Mega JL , Walker JR , Ruff CT , Vandell AG , Nordio F , Deenadayalu N , Murphy SA , Lee J , Mercuri MF , Giugliano RP , Antman EM , Braunwald E and Sabatine MS (2015) Genetics and the clinical response to warfarin and edoxaban: Findings from the randomised, double-blind ENGAGE AF-TIMI 48 trial. Lancet 385(9984), 2280–2287. 25769357 10.1016/S0140-6736(14)61994-2

[r100] Mehta D , Uber R , Ingle T , Li C , Liu Z , Thakkar S , Ning B , Wu L , Yang J , Harris S , Zhou G , Xu J , Tong W , Lesko L and Fang H (2020) Study of pharmacogenomic information in FDA-approved drug labeling to facilitate application of precision medicine. Drug Discovery Today 25(5), 813–820. 32032705 10.1016/j.drudis.2020.01.023

[r101] Mitropoulou C , Fragoulakis V , Bozina N , Vozikis A , Supe S , Bozina T , Poljakovic Z , van Schaik RH and Patrinos GP (2015) Economic evaluation of pharmacogenomic-guided warfarin treatment for elderly Croatian atrial fibrillation patients with ischemic stroke. Pharmacogenomics 16(2), 137–148. 25616100 10.2217/pgs.14.167

[r102] Overby CL , Erwin AL , Abul-Husn NS , Ellis SB , Scott SA , Obeng AO , Kannry JL , Hripcsak G , Bottinger EP and Gottesman O (2014) Physician attitudes toward adopting genome-guided prescribing through clinical decision support. Journal of Personalized Medicine 4(1), 35–49. 25562141 10.3390/jpm4010035 PMC4251406

[r103] Pacanowski MA , Gong Y , Cooper-Dehoff RM , Schork NJ , Shriver MD , Langaee TY , Pepine CJ , Johnson JA and Investigators I (2008) Beta-adrenergic receptor gene polymorphisms and beta-blocker treatment outcomes in hypertension. Clinical Pharmacology and Therapeutics 84(6), 715–721. 18615004 10.1038/clpt.2008.139 PMC2675574

[r104] Pan Y , Chen W , Xu Y , Yi X , Han Y , Yang Q , Li X , Huang L , Johnston SC , Zhao X , Liu L , Zhang Q , Wang G , Wang Y and Wang Y (2017) Genetic polymorphisms and clopidogrel efficacy for acute ischemic stroke or transient ischemic attack: A systematic review and meta-analysis. Circulation 135(1), 21–33. 27806998 10.1161/CIRCULATIONAHA.116.024913

[r105] Pereira NL , Farkouh ME , So D , Lennon R , Geller N , Mathew V , Bell M , Bae JH , Jeong MH , Chavez I , Gordon P , Abbott JD , Cagin C , Baudhuin L , Fu YP , Goodman SG , Hasan A , Iturriaga E , Lerman A , Sidhu M , Tanguay JF , Wang L , Weinshilboum R , Welsh R , Rosenberg Y , Bailey K and Rihal C (2020) Effect of genotype-guided oral P2Y12 inhibitor selection vs conventional clopidogrel therapy on ischemic outcomes after percutaneous coronary intervention: The TAILOR-PCI randomized clinical trial. JAMA 324(8), 761–771. 32840598 10.1001/jama.2020.12443 PMC7448831

[r106] Perera MA , Cavallari LH , Limdi NA , Gamazon ER , Konkashbaev A , Daneshjou R , Pluzhnikov A , Crawford DC , Wang J , Liu N , Tatonetti N , Bourgeois S , Takahashi H , Bradford Y , Burkley BM , Desnick RJ , Halperin JL , Khalifa SI , Langaee TY , Lubitz SA , Nutescu EA , Oetjens M , Shahin MH , Patel SR , Sagreiya H , Tector M , Weck KE , Rieder MJ , Scott SA , Wu AH , Burmester JK , Wadelius M , Deloukas P , Wagner MJ , Mushiroda T , Kubo M , Roden DM , Cox NJ , Altman RB , Klein TE , Nakamura Y and Johnson JA (2013) Genetic variants associated with warfarin dose in African-American individuals: A genome-wide association study. Lancet 382(9894), 790–796. 23755828 10.1016/S0140-6736(13)60681-9 PMC3759580

[r107] Peterson JF , Field JR , Unertl KM , Schildcrout JS , Johnson DC , Shi Y , Danciu I , Cleator JH , Pulley JM , McPherson JA , Denny JC , Laposata M , Roden DM and Johnson KB (2016) Physician response to implementation of genotype-tailored antiplatelet therapy. Clinical Pharmacology and Therapeutics 100(1), 67–74. 26693963 10.1002/cpt.331 PMC4899238

[r108] Peyser B , Perry EP , Singh K , Gill RD , Mehan MR , Haga SB , Musty MD , Milazzo NA , Savard D , Li YJ , Trujilio G and Voora D (2018) Effects of delivering SLCO1B1 pharmacogenetic information in randomized trial and observational settings. Circulation: Genomic and Precision Medicine 11(9), e002228. 30354330 10.1161/CIRCGEN.118.002228

[r109] PharmGKB (2023a) Drug Label Information and Legend. Available at https://www.pharmgkb.org/page/clinAnnLevels (accessed 20 March 2023).

[r110] PharmGKB (2023b) Very Important Pharmacogenes. Available at https://www.pharmgkb.org/vips (accessed 20 March 2023).

[r111] Pirmohamed M , Burnside G , Eriksson N , Jorgensen AL , Toh CH , Nicholson T , Kesteven P , Christersson C , Wahlstrom B , Stafberg C , Zhang JE , Leathart JB , Kohnke H , Maitland-van der Zee AH , Williamson PR , Daly AK , Avery P , Kamali F , Wadelius M and Group E-P (2013) A randomized trial of genotype-guided dosing of warfarin. New England Journal of Medicine 369(24), 2294–2303. 24251363 10.1056/NEJMoa1311386

[r112] Pokorney SD , Simon DN , Thomas L , Fonarow GC , Kowey PR , Chang P , Singer DE , Ansell J , Blanco RG , Gersh B , Mahaffey KW. , Hylek EM , Go AS , Piccini JP , Peterson ED and Outcomes Registry for Better Informed Treatment of Atrial Fibrillation (2015) Patients’ time in therapeutic range on warfarin among US patients with atrial fibrillation: Results from ORBIT-AF registry. American Heart Journal 170(1), 141–148 e1. 26093875 10.1016/j.ahj.2015.03.017

[r113] Popejoy AB , Crooks KR , Fullerton SM , Hindorff LA , Hooker GW , Koenig BA , Pino N , Ramos EM , Ritter DI , Wand H , Wright MW , Yudell M , Zou JY , Plon SE , Bustamante CD , Ormond KE , Clinical Genome Resource A and Diversity Working G (2020) Clinical genetics lacks standard definitions and protocols for the collection and use of diversity measures. American Journal of Human Genetics 107(1), 72–82. 32504544 10.1016/j.ajhg.2020.05.005 PMC7332657

[r114] Pratt VM , Cavallari LH , Del Tredici AL , Hachad H , Ji Y , Kalman LV , Ly RC , Moyer AM , Scott SA , Whirl-Carrillo M and Weck KE (2020) Recommendations for clinical warfarin genotyping allele selection: A report of the Association for Molecular Pathology and the College of American Pathologists. Journal of Molecular Diagnostics 22(7), 847–859. 10.1016/j.jmoldx.2020.04.204 PMC7722527 32380173

[r115] Pratt VM , Del Tredici AL , Hachad H , Ji Y , Kalman LV , Scott SA and Weck KE (2018) Recommendations for clinical CYP2C19 genotyping allele selection: A report of the Association for Molecular Pathology. Journal of Molecular Diagnostics 20(3), 269–276. 10.1016/j.jmoldx.2018.01.011 29474986

[r116] Price MJ , Murray SS , Angiolillo DJ , Lillie E , Smith EN , Tisch RL , Schork NJ , Teirstein PS , Topol EJ and Investigators G (2012) Influence of genetic polymorphisms on the effect of high- and standard-dose clopidogrel after percutaneous coronary intervention: The GIFT (genotype information and functional testing) study. Journal of the American College of Cardiology 59(22), 1928–1937. 22624833 10.1016/j.jacc.2011.11.068

[r117] Pritchard D , Patel JN , Stephens L and Mc Leod HL (2022) Comparison of FDA Table of Pharmacogenetic Associations and Clinical Pharmacogenetics Implementation Consortium guidelines. American Society of Health-System Pharmacists 79(12), 993–1005. 10.1093/ajhp/zxac064 PMC9171570 35230418

[r118] Proietti M , Romanazzi I , Romiti GF , Farcomeni A and Lip GYH (2018) Real-world use of Apixaban for stroke prevention in atrial fibrillation: A systematic review and meta-analysis. Stroke 49(1), 98–106. 29167388 10.1161/STROKEAHA.117.018395

[r119] Ramsey LB , Johnson SG , Caudle KE , Haidar CE , Voora D , Wilke RA , Maxwell WD , McLeod HL , Krauss RM , Roden DM , Feng Q , Cooper-DeHoff RM , Gong L , Klein TE , Wadelius M and Niemi M (2014) The clinical pharmacogenetics implementation consortium guideline for SLCO1B1 and simvastatin-induced myopathy: 2014 update. Clinical Pharmacology and Therapeutics 96(4), 423–428. 24918167 10.1038/clpt.2014.125 PMC4169720

[r120] Relling MV and Evans WE (2015) Pharmacogenomics in the clinic. Nature 526(7573), 343–350. 26469045 10.1038/nature15817 PMC4711261

[r121] Relling MV , Klein TE , Gammal RS , Whirl-Carrillo M , Hoffman JM and Caudle KE (2020) The Clinical Pharmacogenetics Implementation Consortium: 10 years later. Clinical Pharmacology and Therapeutics 107(1), 171–175. 31562822 10.1002/cpt.1651 PMC6925644

[r122] Relling MV , Schwab M , Whirl-Carrillo M , Suarez-Kurtz G , Pui CH , Stein CM , Moyer AM , Evans WE , Klein TE , Antillon-Klussmann FG , Caudle KE , Kato M , Yeoh AEJ , Schmiegelow K and Yang JJ (2019) Clinical Pharmacogenetics Implementation Consortium guideline for thiopurine dosing based on TPMT and NUDT15 genotypes: 2018 update. Clinical Pharmacology and Therapeutics 105(5), 1095–1105. 30447069 10.1002/cpt.1304 PMC6576267

[r123] Roden DM , Van Driest SL , Mosley JD , Wells QS , Robinson JR , Denny JC and Peterson JF (2018) Benefit of preemptive pharmacogenetic information on clinical outcome. Clinical Pharmacology and Therapeutics 103(5), 787–794. 29377064 10.1002/cpt.1035 PMC6134843

[r124] Roden DM and Viswanathan PC (2005) Genetics of acquired long QT syndrome. Journal of Clinical Investigation 115(8), 2025–2032. 16075043 10.1172/JCI25539 PMC1180553

[r125] Roffi M , Patrono C , Collet JP , Mueller C , Valgimigli M , Andreotti F , Bax JJ , Borger MA , Brotons C , Chew DP , Gencer B , Hasenfuss G , Kjeldsen K , Lancellotti P , Landmesser U , Mehilli J , Mukherjee D , Storey RF , Windecker S and Group ESCSD (2016) 2015 ESC guidelines for the management of acute coronary syndromes in patients presenting without persistent ST-segment elevation: Task force for the management of acute coronary syndromes in patients presenting without persistent ST-segment elevation of the European Society of Cardiology (ESC). European Heart Journal 37(3), 267–315. 26320110 10.1093/eurheartj/ehv320

[r126] Rollini F , Franchi F and Angiolillo DJ (2016) Switching P2Y12-receptor inhibitors in patients with coronary artery disease. Nature Reviews. Cardiology 13(1), 11–27. 26283269 10.1038/nrcardio.2015.113

[r127] Rost S , Fregin A , Ivaskevicius V , Conzelmann E , Hortnagel K , Pelz HJ , Lappegard K , Seifried E , Scharrer I , Tuddenham EG , Muller CR , Strom TM and Oldenburg J (2004) Mutations in VKORC1 cause warfarin resistance and multiple coagulation factor deficiency type 2. Nature 427(6974), 537–541. 14765194 10.1038/nature02214

[r128] Rouini MR and Afshar M (2017) Effect of CYP2D6 polymorphisms on the pharmacokinetics of propafenone and its two main metabolites. Thérapie 72(3), 373–382. 28087064 10.1016/j.therap.2016.10.005

[r129] Saldivar JS , Taylor D , Sugarman EA , Cullors A , Garces JA , Oades K and Centeno J (2016) Initial assessment of the benefits of implementing pharmacogenetics into the medical management of patients in a long-term care facility. Pharmacogenomics and Personalized Medicine 9, 1–6. 26855597 10.2147/PGPM.S93480 PMC4725634

[r130] Sangkuhl K , Klein TE and Altman RB (2010) Clopidogrel pathway. Pharmacogenetics and Genomics 20(7), 463–465. 20440227 10.1097/FPC.0b013e3283385420 PMC3086847

[r131] Scheuner MT , Sales P , Hoggatt K , Zhang N , Whooley MA and Kelley MJ (2023) Genetics professionals are key to the integration of genetic testing within the practice of frontline clinicians. Genetics in Medicine 25(1), 103–114. 36301261 10.1016/j.gim.2022.09.012

[r132] Schoonen WM , Thomas SL , Somers EC , Smeeth L , Kim J , Evans S and Hall AJ (2010) Do selected drugs increase the risk of lupus? A matched case-control study. British Journal of Clinical Pharmacology 70(4), 588–596. 20840450 10.1111/j.1365-2125.2010.03733.x PMC2950993

[r133] Schork NJ (2015) Personalized medicine: Time for one-person trials. Nature 520(7549), 609–611. 25925459 10.1038/520609a

[r134] Scott SA , Sangkuhl K , Stein CM , Hulot JS , Mega JL , Roden DM , Klein TE , Sabatine MS , Johnson JA , Shuldiner AR and Clinical Pharmacogenetics Implementation (2013) Clinical Pharmacogenetics Implementation Consortium guidelines for CYP2C19 genotype and clopidogrel therapy: 2013 update. Clinical Pharmacology and Therapeutics 94(3), 317–323. 23698643 10.1038/clpt.2013.105 PMC3748366

[r135] Shi J , Wang X , Nguyen JH , Bleske BE , Liang Y , Liu L and Zhu HJ (2016) Dabigatran etexilate activation is affected by the CES1 genetic polymorphism G143E (rs71647871) and gender. Biochemical Pharmacology 119, 76–84. 27614009 10.1016/j.bcp.2016.09.003 PMC5061634

[r136] Shitara Y (2011) Clinical importance of OATP1B1 and OATP1B3 in drug-drug interactions. Drug Metabolism and Pharmacokinetics 26(3), 220–227. 21297316 10.2133/dmpk.DMPK-10-RV-094

[r137] Shugg T , Pasternak AL , London B and Luzum JA (2020) Prevalence and types of inconsistencies in clinical pharmacogenetic recommendations among major U.S. sources. NPJGenomic Medicine 5, 48. 10.1038/s41525-020-00156-7 PMC7603298 33145028

[r138] Smith DM , Weitzel KW , Elsey AR , Langaee T , Gong Y , Wake DT , Duong BQ , Hagen M , Harle CA , Mercado E , Nagoshi Y , Newsom K , Wright A , Rosenberg EI , Starostik P , Clare-Salzler MJ , Schmidt SO , Fillingim RB , Johnson JA and Cavallari LH (2019) CYP2D6-guided opioid therapy improves pain control in CYP2D6 intermediate and poor metabolizers: A pragmatic clinical trial. Genetics in Medicine 21(8), 1842–1850. 30670877 10.1038/s41436-018-0431-8 PMC6650382

[r139] Sorich MJ , Rowland A , McKinnon RA and Wiese MD (2014) CYP2C19 genotype has a greater effect on adverse cardiovascular outcomes following percutaneous coronary intervention and in Asian populations treated with clopidogrel: A meta-analysis. Circulation. Cardiovascular Genetics 7(6), 895–902. 25258374 10.1161/CIRCGENETICS.114.000669

[r140] Spinasse LB , Santos AR , Suffys PN , Muxfeldt ES and Salles GF (2014) Different phenotypes of the NAT2 gene influences hydralazine antihypertensive response in patients with resistant hypertension. Pharmacogenomics 15(2), 169–178. 24444407 10.2217/pgs.13.202

[r141] Stanek EJ , Sanders CL , Taber KA , Khalid M , Patel A , Verbrugge RR , Agatep BC , Aubert RE , Epstein RS and Frueh FW (2012) Adoption of pharmacogenomic testing by US physicians: Results of a nationwide survey. Clinical Pharmacology and Therapeutics 91(3), 450–458. 22278335 10.1038/clpt.2011.306

[r142] Strauss DG , Vicente J , Johannesen L , Blinova K , Mason JW , Weeke P , Behr ER , Roden DM , Woosley R , Kosova G , Rosenberg MA and Newton-Cheh C (2017) Common genetic variant risk score is associated with drug-induced QT prolongation and torsade de pointes risk: A pilot study. Circulation 135(14), 1300–1310. 28213480 10.1161/CIRCULATIONAHA.116.023980 PMC5380476

[r143] Sukri A , Salleh MZ , Masimirembwa C and Teh LK (2022) A systematic review on the cost effectiveness of pharmacogenomics in developing countries: Implementation challenges. Pharmacogenomics Journal 22(3), 147–159. 35319010 10.1038/s41397-022-00272-w

[r144] Swen JJ , van der Wouden CH , Manson LE , Abdullah-Koolmees H , Blagec K , Blagus T , Bohringer S , Cambon-Thomsen A , Cecchin E , Cheung KC , Deneer VH , Dupui M , Ingelman-Sundberg M , Jonsson S , Joefield-Roka C , Just KS , Karlsson MO , Konta L , Koopmann R , Kriek M , Lehr T , Mitropoulou C , Rial-Sebbag E , Rollinson V , Roncato R , Samwald M , Schaeffeler E , Skokou M , Schwab M , Steinberger D , Stingl JC , Tremmel R , Turner RM , van Rhenen MH , Davila Fajardo CL , Dolzan V , Patrinos GP , Pirmohamed M , Sunder-Plassmann G , Toffoli G and Guchelaar HJ (2023) A 12-gene pharmacogenetic panel to prevent adverse drug reactions: An open-label, multicentre, controlled, cluster-randomised crossover implementation study. Lancet 401(10374), 347–356. 36739136 10.1016/S0140-6736(22)01841-4

[r145] Tata EB , Ambele MA and Pepper MS (2020) Barriers to implementing clinical pharmacogenetics testing in Sub-Saharan Africa. A critical review. Pharmaceutics 12(9), 809. 32858798 10.3390/pharmaceutics12090809 PMC7560181

[r146] Tayeh MK , Gaedigk A , Goetz MP , Klein TE , Lyon E , McMillin GA , Rentas S , Shinawi M , Pratt VM , Scott SA and ACMG Laboratory Quality Assurance Committee (2022) Clinical pharmacogenomic testing and reporting: A technical standard of the American College of Medical Genetics and Genomics (ACMG). Genetics in Medicine 24(4), 759–768. 35177334 10.1016/j.gim.2021.12.009

[r147] Tirona RG , Leake BF , Merino G and Kim RB (2001) Polymorphisms in OATP-C: Identification of multiple allelic variants associated with altered transport activity among European- and African-Americans. Journal of Biological Chemistry 276(38), 35669–35675. 11477075 10.1074/jbc.M103792200

[r148] Turner RM , Fontana V , Zhang JE , Carr D , Yin P , FitzGerald R , Morris AP and Pirmohamed M (2020) A genome-wide association study of circulating levels of atorvastatin and its major metabolites. Clinical Pharmacology and Therapeutics 108(2), 287–297. 32128760 10.1002/cpt.1820

[r149] Turner RM and Pirmohamed M (2019) Statin-related myotoxicity: A comprehensive review of pharmacokinetic, pharmacogenomic and muscle components. Journal of Clinical Medicine 9(1), 22. 31861911 10.3390/jcm9010022 PMC7019839

[r150] Turongkaravee S , Jittikoon J , Rochanathimoke O , Boyd K , Wu O and Chaikledkaew U (2021) Pharmacogenetic testing for adverse drug reaction prevention: Systematic review of economic evaluations and the appraisal of quality matters for clinical practice and implementation. BMC Health Services Research 21(1), 1042. 34600523 10.1186/s12913-021-07025-8 PMC8487501

[r151] van der Wouden CH , Cambon-Thomsen A , Cecchin E , Cheung KC , Davila-Fajardo CL , Deneer VH , Dolzan V , Ingelman-Sundberg M , Jonsson S , Karlsson MO , Kriek M , Mitropoulou C , Patrinos GP , Pirmohamed M , Samwald M , Schaeffeler E , Schwab M , Steinberger D , Stingl J , Sunder-Plassmann G , Toffoli G , Turner RM , van Rhenen MH , Swen JJ , Guchelaar HJ and Ubiquitous Pharmacogenomics (2017) Implementing pharmacogenomics in Europe: Design and implementation strategy of the ubiquitous pharmacogenomics consortium. Clinical Pharmacology and Therapeutics 101(3), 341–358. 28027596 10.1002/cpt.602

[r152] Varenhorst C , James S , Erlinge D , Brandt JT , Braun OO , Man M , Siegbahn A , Walker J , Wallentin L , Winters KJ and Close SL (2009) Genetic variation of CYP2C19 affects both pharmacokinetic and pharmacodynamic responses to clopidogrel but not prasugrel in aspirin-treated patients with coronary artery disease. European Heart Journal 30(14), 1744–1752. 19429918 10.1093/eurheartj/ehp157 PMC2709885

[r153] Verhoef TI , Redekop WK , Daly AK , van Schie RM , de Boer A and Maitland-van der Zee AH (2014) Pharmacogenetic-guided dosing of coumarin anticoagulants: Algorithms for warfarin, acenocoumarol and phenprocoumon. British Journal of Clinical Pharmacology 77(4), 626–641. 23919835 10.1111/bcp.12220 PMC3971980

[r154] Vieira CP , Neves DV , Coelho EB and Lanchote VL (2018) Effect of CYP2D6 poor metabolizer phenotype on stereoselective nebivolol pharmacokinetics. Drug Metabolism Letters 12(1), 68–70. 29676238 10.2174/1872312812666180420104945

[r155] Voora D , Shah SH , Spasojevic I , Ali S , Reed CR , Salisbury BA and Ginsburg GS (2009) The SLCO1B1*5 genetic variant is associated with statin-induced side effects. Journal of the American College of Cardiology 54(17), 1609–1616. 19833260 10.1016/j.jacc.2009.04.053 PMC3417133

[r156] Wada Y , Yang T , Shaffer CM , Daniel LL , Glazer AM , Davogustto GE , Lowery BD , Farber-Eger EH , Wells QS and Roden DM (2022) Common ancestry-specific ion channel variants predispose to drug-induced arrhythmias. Circulation 145(4), 299–308. 34994586 10.1161/CIRCULATIONAHA.121.054883 PMC8852297

[r157] Wadelius M , Chen LY , Lindh JD , Eriksson N , Ghori MJ , Bumpstead S , Holm L , McGinnis R , Rane A and Deloukas P (2009) The largest prospective warfarin-treated cohort supports genetic forecasting. Blood 113(4), 784–792. 18574025 10.1182/blood-2008-04-149070 PMC2630264

[r158] Wallentin L , Becker RC , Budaj A , Cannon CP , Emanuelsson H , Held C , Horrow J , Husted S , James S , Katus H , Mahaffey KW , Scirica BM , Skene A , Steg PG , Storey RF , Harrington RA , Investigators P , Freij A and Thorsen M (2009) Ticagrelor versus clopidogrel in patients with acute coronary syndromes. New England Journal of Medicine 361(11), 1045–1057. 19717846 10.1056/NEJMoa0904327

[r159] Wallentin L , James S , Storey RF , Armstrong M , Barratt BJ , Horrow J , Husted S , Katus H , Steg PG , Shah SH , Becker RC and investigators P (2010) Effect of CYP2C19 and ABCB1 single nucleotide polymorphisms on outcomes of treatment with ticagrelor versus clopidogrel for acute coronary syndromes: A genetic substudy of the PLATO trial. Lancet 376(9749), 1320–1328. 20801498 10.1016/S0140-6736(10)61274-3

[r160] Wang Y , Zhao X , Lin J , Li H , Johnston SC , Lin Y , Pan Y , Liu L , Wang D , Wang C , Meng X , Xu J , Wang Y and investigators C (2016) Association between CYP2C19 loss-of-function allele status and efficacy of clopidogrel for risk reduction among patients with minor stroke or transient ischemic attack. JAMA 316(1), 70–78. 27348249 10.1001/jama.2016.8662

[r161] Weber WW and Hein DW (1985) N-acetylation pharmacogenetics. Pharmacological Reviews 37(1), 25–79. 2860675

[r162] Whelton PK , Carey RM , Aronow WS , Casey DE , Collins KJ , Dennison Himmelfarb C , DePalma SM , Gidding S , Jamerson KA , Jones DW , MacLaughlin EJ , Muntner P , Ovbiagele B , Smith SC , Spencer CC , Stafford RS , Taler SJ , Thomas RJ , Williams KA Sr , Williamson JD and Wright JT Jr (2018) 2017 ACC/AHA/AAPA/ABC/ACPM/AGS/APhA/ASH/ASPC/NMA/PCNA guideline for the prevention, detection, evaluation, and management of high blood pressure in adults: Executive summary: A report of the American College of Cardiology/American Heart Association task force on clinical practice guidelines. Circulation 138(17), e426–e483. 30354655 10.1161/CIR.0000000000000597

[r163] White HL , de Boer RA , Maqbool A , Greenwood D , van Veldhuisen DJ , Cuthbert R , Ball SG , Hall AS , Balmforth AJ and Group M-HS (2003) An evaluation of the beta-1 adrenergic receptor Arg389Gly polymorphism in individuals with heart failure: A MERIT-HF sub-study. European Journal of Heart Failure 5(4), 463–468. 12921807 10.1016/s1388-9842(03)00044-8

[r164] Winner JG , Carhart JM , Altar CA , Goldfarb S , Allen JD , Lavezzari G , Parsons KK , Marshak AG , Garavaglia S and Dechairo BM (2015) Combinatorial pharmacogenomic guidance for psychiatric medications reduces overall pharmacy costs in a 1 year prospective evaluation. Current Medical Research and Opinion 31(9), 1633–1643. 26086890 10.1185/03007995.2015.1063483

[r165] Zabalza M , Subirana I , Sala J , Lluis-Ganella C , Lucas G , Tomas M , Masia R , Marrugat J , Brugada R and Elosua R (2012) Meta-analyses of the association between cytochrome CYP2C19 loss- and gain-of-function polymorphisms and cardiovascular outcomes in patients with coronary artery disease treated with clopidogrel. Heart 98(2), 100–108. 21693476 10.1136/hrt.2011.227652

[r166] Zhou Y and Lauschke VM (2022) Population pharmacogenomics: An update on ethnogeographic differences and opportunities for precision public health. Human Genetics 141(6), 1113–1136. 34652573 10.1007/s00439-021-02385-x PMC9177500

[r167] Zhou Y , Nevosadova L , Eliasson E and Lauschke VM (2023) Global distribution of functionally important CYP2C9 alleles and their inferred metabolic consequences. Human Genomics 17(1), 15. 36855170 10.1186/s40246-023-00461-z PMC9976394

[r168] Zhu J , Alexander GC , Nazarian S , Segal JB and Wu AW (2018) Trends and variation in oral anticoagulant choice in patients with atrial fibrillation, 2010–2017. Pharmacotherapy 38(9), 907–920. 29920705 10.1002/phar.2158 PMC6448138

[r169] Zhu Y , Swanson KM , Rojas RL , Wang Z , St Sauver JL , Visscher SL , Prokop LJ , Bielinski SJ , Wang L , Weinshilboum R and Borah BJ (2020) Systematic review of the evidence on the cost-effectiveness of pharmacogenomics-guided treatment for cardiovascular diseases. Genetics in Medicine 22(3), 475–486. 31591509 10.1038/s41436-019-0667-y PMC7056639

[r170] Zisaki A , Miskovic L and Hatzimanikatis V (2015) Antihypertensive drugs metabolism: An update to pharmacokinetic profiles and computational approaches. Current Pharmaceutical Design 21(6), 806–822. 25341854 10.2174/1381612820666141024151119 PMC4435036

