# Supplementary material for: Cardiovascular precision medicine – A pharmacogenomic perspective
Source: Camb Prism Precis Med. 2023 Jun 29;1:e28. doi: 10.1017/pcm.2023.17 (PMC10953758; doi:10.1017/pcm.2023.17)
Supplement: Peer review history

# Reviewer report: Review: Cardiovascular precision medicine – A pharmacogenomic perspective — R0/PR2

**DOI:** 10.1017/pcm.2023.17.pr2

Title: Cardiovascular precision medicine – a pharmacogenomic perspective

Authors: Sandosh Padmanabhan et al

Although there are many published reviews on the pharmacogenetics and pharmacogenomics of drugs used in cardiovascular diseases, I found this review to be largely comprehensive, up to date and concise. However, I believe that the readers would benefit from including a section on future perspectives in CVD pharmacogenomics including approaches to expedite the implementation of PGx in diverse healthcare systems (i.e. in developed and developing countries).

The global geographical distribution of alleles and phenotypes of most drug metabolizing enzymes have been covered extensively in the literature (PMID: 34652573, PMID: 36855170 as examples) and I am therefore wondering if figures 1-3 are necessary? Are the data presented here presenting anything new or distinct from what have been published?

Minor point

On page 5, the authors state “ …. pharmacogenomics (PGx), the study of the effect of inherited genetic variation on drug….” I believe that acquired variations are also relevant to pharmacogenomics.

# Editor report: Recommendation: Cardiovascular precision medicine – A pharmacogenomic perspective — R0/PR3

**DOI:** 10.1017/pcm.2023.17.pr3

**Affiliations:** Vifor Pharma Ltd, Switzerland

No accompanying comment.

# Editor report: Decision: Cardiovascular precision medicine – A pharmacogenomic perspective — R0/PR4

**DOI:** 10.1017/pcm.2023.17.pr4

**Affiliations:** Shanghai Institute of Hypertension, China

No accompanying comment.

# Reviewer report: Review: Cardiovascular precision medicine – A pharmacogenomic perspective — R1/PR6

**DOI:** 10.1017/pcm.2023.17.pr6

The authors have addressed my previous comments adequately.

# Editor report: Recommendation: Cardiovascular precision medicine – A pharmacogenomic perspective — R1/PR7

**DOI:** 10.1017/pcm.2023.17.pr7

**Affiliations:** Vifor Pharma Ltd, Switzerland

No accompanying comment.

# Editor report: Decision: Cardiovascular precision medicine – A pharmacogenomic perspective — R1/PR8

**DOI:** 10.1017/pcm.2023.17.pr8

**Affiliations:** Shanghai Institute of Hypertension, China

No accompanying comment.
